# Factors impacting protective immunity conferred by vaccination with adeno-associated virus-like particles displaying human papillomavirus L2 residues 17–36

**DOI:** 10.1128/jvi.00448-26

**Published:** 2026-08-21

**Authors:** Jade Alvarez, Pola Olczak, Hua-Ling Tsai, Jasmine Terrell, Margaret Wong, Hannah Tsingine, Vikrant Palande, Jeanette Prangsgaard, Emma Huus, Martin Műller, John Dirk Vestergaard Nieland, Richard B. S. Roden

**Affiliations:** 1 Department of Pathology, Johns Hopkins School of Medicine 1500 , Baltimore, Maryland, USA; 2 Department of Biostatistics, Johns Hopkins School of Medicine 1500 , Baltimore, Maryland, USA; 3 2A Pharma ApS, Aalborg, Denmark; 4 Deutsches Krebsforschungszentrum 28333, Heidelberg, Germany; 5 Aalborg University 1004, Gistrup, Denmark; 6 Department of Oncology, Johns Hopkins School of Medicine 1500 , Baltimore, Maryland, USA; 7 Department of Gynecology/Obstetrics, Johns Hopkins School of Medicine 1500 , Baltimore, Maryland, USA; Tufts University, Boston, Massachusetts, USA

**Keywords:** Adeno-associated virus, AAV2, neutralizing antibody, L2, prophylactic vaccine, HPV, human papillomavirus

## Abstract

A phase I study was conducted in 20 healthy subjects with 20 µg adeno-associated virus 2 (AAV2)-like particles displaying L2 residues 17–36 of both human papillomavirus (HPV) type 16 and HPV31 (AAVLP-HPV) or placebo. AAVLP-HPV vaccination was well tolerated and induced a modest L2-specific serum antibody titer that neutralizes HPV16, HPV31, and HPV5 *in vitro*. Here, we show that upon passive transfer into naïve mice, the sera from a subset of patients vaccinated with AAVLP-HPV conferred protection against HPV16, HPV31, and HPV5, but not HPV76. HPV16-naïve patients required three AAVLP-HPV doses to develop protection, whereas previously exposed individuals achieved protection after two doses and exhibited higher L2-specific and neutralizing antibody titers. The avidity of the L2-specific antibody response was low, but broadly cross-reactive. Prior exposure to AAV2 compromised the strength and duration of the L2 neutralizing antibody response, as well as protective immunity against HPV16, suggesting original antigenic sin. AAVLP-HPV induced high AAV2 neutralizing titers after one vaccination in AAV2-exposed or three in AAV2-naive patients. Thus, prior HPV and AAV2 infections impact the interpretation of AAVLP-HPV immunogenicity.

**IMPORTANCE:**

HPV is among the most prevalent sexually transmitted infections. Persistent infection with a high-risk genotype (hrHPV) can cause cervical and other anogenital, and oropharyngeal cancers. There are a dozen hrHPV genotypes, HPV16 being the dominant type in these cancers; only ≤7 are targeted by licensed HPV vaccines. Ten intermediate-risk genotypes are also found in small proportions of cervical cancers. HPV6 and HPV11, while commonly associated with benign genital warts, can be carcinogenic in humans. The HPV+ cancer burden is highest in developing countries where cervical screening and vaccination rates are low, resulting in >500,000 cervical cancer cases annually. The plethora of skin-tropic HPVs are transmitted non-sexually and generally cause benign, self-limiting infections, but a subset of *betapapillomavirus*es is associated with cutaneous squamous cell carcinoma in epidermodysplasia verruciformis and immunocompromised patients. A simple, low-cost vaccine preventing infection by all of these medically significant HPV types is needed.

**CLINICAL TRIALS:**

This study is registered with ClinicalTrials.gov as NCT03929172.

## INTRODUCTION

The current licensed vaccines, based on virus-like particles (VLP) of the L1 major capsid protein, induce a strong but type-specific immunity, e.g., Gardasil9, which targets seven hrHPV (types 16, 18, 31, 33, 45, 52, and 58) and two benign genital wart types, HPV6/11 (1). Passive transfer of L1 VLP-specific immune sera or IgG to naïve animals confers type-restricted protection against cutaneous or mucosal challenge with native animal papillomavirus models (2–4), HPV quasivirions containing CRPV genomes (5), or HPV pseudovirions (PsV) delivering a luciferase reporter (6). L1-specific neutralizing antibodies bind to the closely arrayed epitopes on the capsid with high affinity (7). Infection in all these experimental models requires microtrauma of the epithelium, which presumably triggers exudation, allowing serum IgG to neutralize the viral inoculum (8).

L2-based vaccines have the potential to broaden protection as an alternative to simply increasing L1 VLP vaccine valency (9). Vaccination with L2, particularly targeting conserved protective epitopes, e.g., residues 17–36, can elicit cross-neutralizing antibodies (10–13). Passive immunization with L2 antisera or purified MAbs is also protective against experimental challenge (12, 14, 15). Potentially, a single L2-based vaccine antigen could cover all oncogenic HPV types as well as those associated with cutaneous disease (9). However, a challenge to the development of L2-based vaccines is their weak immunogenicity compared to L1, in part because it does not form a VLP and is mostly buried below the capsid surface (9, 10).

Display of key L2 protective epitopes in immunodominant sites on the surface of a heterologous virus-like particle is a potential approach to enhance the strength and durability of the response. To this end, an adeno-associated virus-like particle (AAVLP) comprising VP3 from AAV2 and displaying both the L2 17–36 epitopes of HPV16 and HPV31 in different VP3 surface loops was developed (16), and the neutralizing antibody and protective responses to vaccination, either alone or in combination with various adjuvants, were tested in mice and rabbits (17). The use of adjuvant significantly increased L2-specific neutralizing antibodies and protection against vaginal challenge of mice with HPV16 (17). However, AAVLP-HPV vaccination of rabbits, even without adjuvant, broadly protected against cutaneous challenge with HPV quasivirions, suggesting that an adjuvant might not be required (17).

In a 12-month, single-center, randomized, placebo-controlled, double-blinded phase 1 clinical trial (NCT03929172), healthy participants (*n* = 20; 8 females/12 males) received three doses of either 20 µg AAVLP-HPV without adjuvant (*n* = 16) or placebo (*n* = 4) on days 1, 57, and 180. The vaccine was well tolerated and induced both HPV16- and HPV31-neutralizing and L2 peptide-specific antibody responses (18). However, these responses were variable, and it is unclear whether they are protective. Here, we sought to determine whether AAVLP-HPV vaccination induced protective immunity in trial participants, as measured by passive transfer studies, and define host factors that influence these responses.

## RESULTS

### Analysis of protective serum antibody responses in study participants

**Fig 1:**
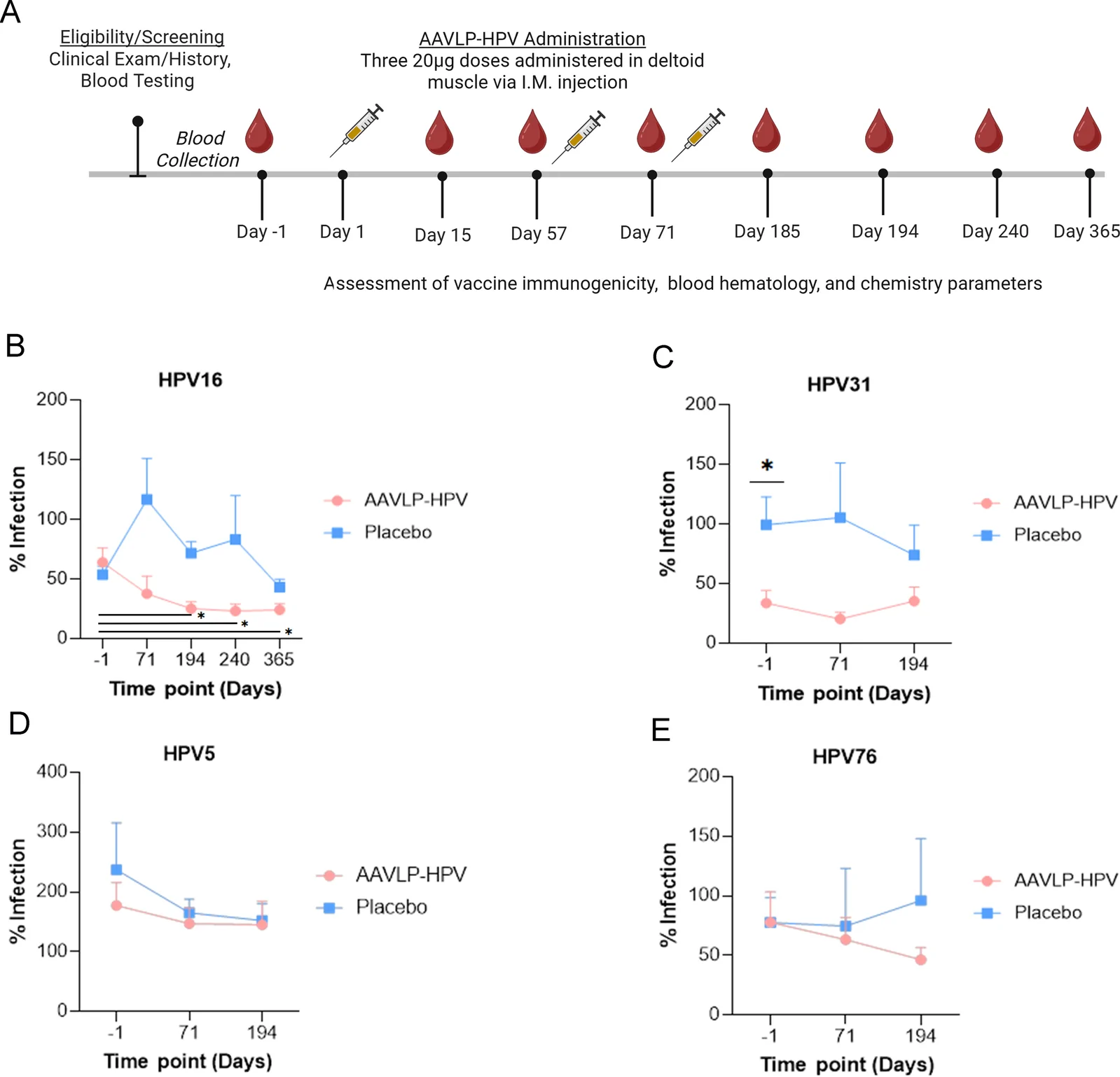
Protective responses conferred by sera from AAVLP-HPV phase I study participants. Naïve CD1 mice received 100 µL of sera collected at the indicated time points from active (*n* = 14) or placebo (*n* = 4) participants in the AAVLP-HPV clinical trial (NCT03929172) (**A**). Mice were challenged with (**B**) HPV16, (**C**) HPV31, (**D**) HPV5, or (**E**) HPV76 PsV 1 day after serum transfer, and infection was assessed by luminescence imaging 3 days later. Percent infection was calculated by normalizing to infection levels observed in mice that did not receive passive transfer of human sera. Mean and standard error of the mean (SEM) are shown. The *P*-values shown were obtained using a mixed-effects model to test for differences in changes from baseline, followed by post hoc comparisons at each time point. *P*-values were corrected for multiple comparisons using the Benjamini-Hochberg method (19). *P < 0.05.

Sera study participants (*n* = 20) who received three doses of either placebo (formulation buffer; *n* = 4) or AAVLP-HPV vaccination (*n* = 14 of 16 total, as two withdrew consent at the beginning of the study), were collected (Fig. 1A) and assessed in mice for protective activity by passive transfer against vaginal challenge with HPV pseudovirus (PsV) delivering the luciferase reporter gene. Female CD1 mice received 100 µL of participant serum via intraperitoneal injection, followed 1 day later by vaginal challenge with HPV16 (4 mice/serum sample) or HPV31, HPV5, or HPV76 (each 1 mouse/serum sample) PsV. Infection was assessed by imaging 3 days post-challenge, and the signal was normalized against mice that received no serum. Participant serum samples collected at baseline (day −1), day 71, and day 194, 2 weeks after the first and second booster doses, respectively, as well as days 240 and 365, were tested. As a positive control, rabbit antiserum to Gardasil consistently provided an average of 99.8% protection against HPV16 and HPV31 challenges, while rabbit antiserum to HPV5 and HPV76 L1 VLPs conferred 99.8% protection against their respective genotypes.

The active treatment group differed significantly from the placebo group over time for both HPV16 and HPV31 (P_gp diff_ = 0.004 and 0.001, respectively, using mixed-effects models to account for within-participant correlations). Here, we define 100% infection as the mean signal obtained after challenge of mice that received no other intervention, indicating 0% protection. Comparing protection by patient sera longitudinally from baseline, those in the AAVLP-HPV vaccinated group developed evidence of increased protection, as indicated by lower percent HPV16 infection at each time point after baseline (Fig. 1B). By contrast, the placebo group did not show a trend in change from baseline level of protection, with apparent variability in the assay and in protection at baseline. Overall change from baseline in protection against HPV16 challenge was significantly greater in the active group compared with placebo (P_gp diff from D-1_ = 0.03, using mixed-effects models and the Benjamini-Hochberg correction to account for multiple comparisons). Compared to baseline, protection was enhanced at days 194 (*P* = 0.03), 240 (*P* = 0.03), and 365 (*P* = 0.03). Protection against HPV31 challenge was greater in the active group compared to placebo, although it showed minimal change over time compared to baseline and likely reflected pre-existing HPV31 neutralizing activity in two of 14 participants (Fig. 1C; Table 1). For HPV types 5 and 76, there was no significant difference between active and placebo groups or trends from baseline, suggesting no cross-protection against these two cutaneous types (Fig. 1C through E).

**TABLE 1:** Baseline serum neutralizing antibody titers for common low- and high-risk HPV genotypes and AAV2^*a*^

ID	HPV 2	HPV 3	HPV 5*	HPV 6*	HPV 11	HPV 16*	HPV 18	HPV 31*	HPV 33	HPV 35	HPV 38*	HPV 39	HPV 45	HPV 51	HPV 52*	HPV 58	HPV 59	HPV 68	HPV 73	HPV 92	AAV2
1	91	70	–	–	–	–	468	–	57	–	–	–	–	–	–	181	–	–	114	–	11
2^*b*^	67	–	–	98	–	–	–	–	–	–	115	–	–	–	–	220	–	–	86	–	413
3	52	–	–	–	–	498	313	–	–	–	–	–	–	–	–	–	–	–	79	–	–
4	–	–	–	151	–	–	–	–	–	–	–	–	–	–	215	–	–	–	62	–	–
5	55	–	–	–	–	–	–	–	–	–	–	–	–	–	–	–	–	–	79	–	–
6	–	–	–	158	–	–	–	1,183	156	**12,000**	–	–	–	121	170	864	–	419	74	–	–
8^*b*^	–	50	–	–	–	–	–	–	–	–	–	–	–	–	–	74	–	–	–	–	195
10	106	125	–	–	–	–	–	–	–	–	–	–	–	–	–	80	–	–	139	–	–
11	51	62	–	171	–	174	–	222	–	–	–	–	–	80	–	217	–	–	112	–	–
12	76	63	–	140	–	–	–	92	–	–	–	270	–	125	–	88	–	–	–	–	–
13	51	59	–	–	–	–	–	–	–	–	–	–	–	–	–	–	–	–	92	–	–
14^*b*^	57	89	–	68	–	–	–	–	–	–	–	–	–	–	–	–	–	–	88	–	58
15	50	–	–	–	–	188	–	–	–	–	–	–	–	–	–	–	–	–	–	–	34
16^*b*^	71	138	–	852	–	–	–	–	–	–	–	–	–	–	–	–	–	–	102	–	262
17	67	107	–	88	–	–	186	86	149	–	–	–	–	–	88	167	292	–	128	–	52
18	70	101	–	349	88	976	2,881	147	1,195	–	79	310	206	–	–	–	–	–	–	–	586
19	173	131	–	166	–	–	–	–	–	–	–	–	–	–	–	–	331	–	102	–	–
20	227	82	–	–	–	–	–	1,516	–	–	–	–	–	266	851	4,027	–	–	71	–	–

^*a*^ For baseline (day −1) serum samples for each participant (listed by identifier [ID]), neutralization IC_50_ titers are shown for the indicated HPV genotypes or AAV2 (far right), each assessed using a pseudovirion-based neutralization assay (PBNA) or furin-cleaved pseudovirion-based neutralization assay (fcPBNA) indicated by an asterisk (*). A dash (–) denotes no measurable titers; for emphasis, shading in the body of the table denotes titers of >100, underlining denotes titers of >1,000, and boldface denotes titers of >10,000.

^*b*^ Subjects 2, 8, 14, and 16 received placebo.

### Assessment of baseline serostatus

The partial protection against HPV16 and HPV31 evident upon passive transfer of baseline sera suggested that natural exposure may have induced protective antibodies in a subset of participants prior to study entry. To assess participants’ baseline HPV serostatus, sera were evaluated for *in vitro* neutralizing antibodies against common genotypes using a pseudovirion-based neutralization assay (PBNA). Natural infections typically trigger only L1-specific and type-restricted neutralizing antibodies, although they can elicit L2-specific antibodies at a low frequency as well. The conventional PBNA has low sensitivity toward L2-specific neutralizing antibodies. Therefore, we selected to test participant sera for HPV16 and HPV31 neutralizing antibodies with the furin-cleaved (fc) PBNA, which detects these responses with greater sensitivity.

Neutralizing activity against HPV16 was detected in the baseline sera of four of 20 participants, and against HPV31 in six, with two sera neutralizing both types (Table 1). Furthermore, neutralizing activity was also detected against other high-risk types, including HPV52 (*n* = 4/20) by the fcPBNA. Neutralizing activity against another nine hrHPV types and one intermediate-risk type (HPV73) was detected by the conventional PBNA (Table 1), suggesting that the unexpectedly high rates of positivity were not due to the increased sensitivity of the fcPBNA. Neutralizing activity against HPV6 was present in baseline sera of 10/20 participants, but only 1/20 had activity against HPV11, consistent with the lower prevalence of the latter type in genital warts. The average age of the eight female and 12 male participants was 34.3 (range 22–45) years, and this level of hrHPV seropositivity suggests substantial prior sexual exposure within this cohort.

Most individuals showed detectable levels of neutralizing antibodies against cutaneous HPV types, such as the common wart types HPV2 and HPV3, typically contracted during childhood (Table 1). By contrast, none of the baseline sera neutralized the betapapillomaviruses HPV5 or HPV92.

Collectively, these findings indicate that individuals in both the active and placebo groups had prior exposure to multiple HPV types. The presence of neutralizing and potentially protective antibodies in baseline sera should be considered when interpreting vaccine-specific immunogenicity and protective responses.

### Impact of prior HPV16/31 neutralizing antibodies on vaccine-induced protective responses

**Fig 2:**
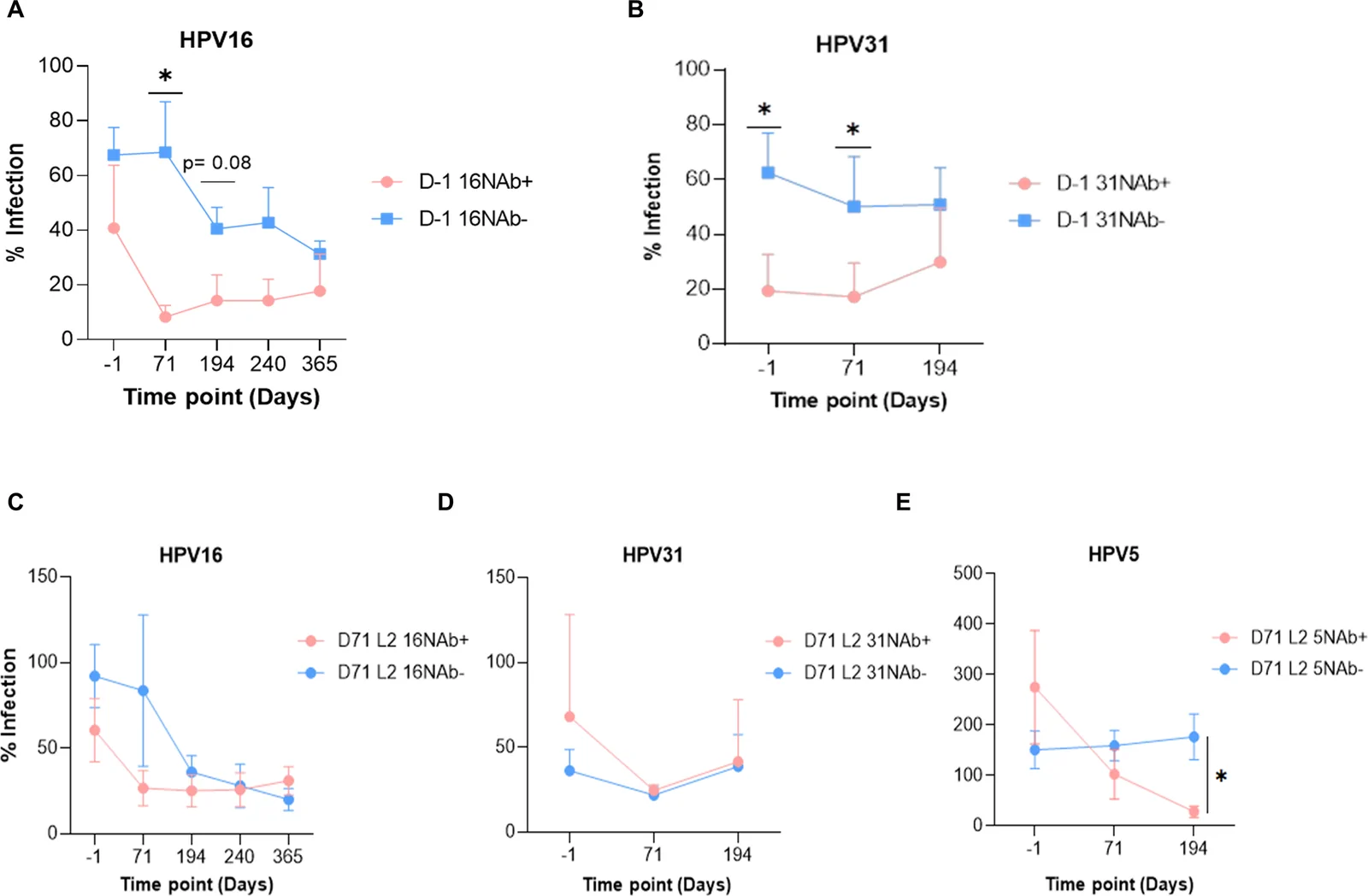
Neutralizing antibody responses in AAVLP-HPV–vaccinated participants. Participants who received AAVLP-HPV vaccination were stratified based on (**A**) whether a pre-existing HPV16 neutralizing antibody titer (*n* = 4) was detected at baseline or not (D-1 16NAb+ vs D-1 16NAb−) and analyzed for their protective response against HPV16 or (**B**) whether a pre-existing HPV31 neutralizing antibody titer (*n* = 6) was detected at baseline or not (D-1 31NAb+ vs D-1 31NAb−) and analyzed for their protective response against HPV31. After omission of subjects with HPV16 neutralizing antibodies at baseline, the AAVLP-HPV vaccine group was stratified based on whether a neutralizing antibody response was detected on day 71 (presumed to be L2-specific and thus labeled D71 L2 NAb+) or not (D71 L2 NAb−). Protective efficacy was then compared against (**C**) HPV16, (**D**) HPV31, and (**E**) HPV5 at each time point. Sample sizes were *n* = 6 for the D71 L2 16NAb+ group and *n* = 2 for the D71 L2 31NAb+ group. No subjects had HPV5 neutralizing antibodies at baseline (*n* = 3 for the D71 L2 5NAb+ group). Data are shown as mean ± SEM. The *P*-values shown were obtained using the Wilcoxon rank-sum test at each time point after first assessing overall group differences and differences in change from baseline using a mixed-effects model. *P < 0.05.

We compared levels of protection against HPV16 or HPV31 conferred by passive transfer of baseline (day −1) sera from participants with detectable neutralizing antibodies versus those without detectable antibodies. Sera from participants with detectable HPV16 neutralizing antibodies in baseline sera (D-1 16NAb+) exhibited an overall greater protection (P_gp diff_ = 0.03) than those without (D-1 16NAb−), although no significant difference in change from baseline between groups was observed (P_gp diff in change from D-1_ = 0.64) (Fig. 2A). Greater protection at day 71 was evident for D-1 16NAb+ participants (Fig. 2A), suggestive of a more rapid response in those with prior exposure to HPV16. Interestingly, this level of protection was maintained for the rest of the study in the group previously exposed to HPV16. For HPV16-naïve patients, the difference closed by day 194, and both groups remained stable through the end of the study.

Sera from participants with detectable HPV31 neutralizing antibodies in baseline sera (D-1 31NAb+) tended toward greater protection (P_gp diff_ = 0.09) than those without (D-1 31NAb−), although protection was incomplete and not significantly different from baseline (P_gp diff in change from D-1_ = 0.76) (Fig. 2B). However, greater protection at days −1 and 71 was evident for D-1 31NAb+ (Fig. 2B), suggestive of a prior protective response to HPV31 that changed little after vaccination. Possibly, the response to the HPV31 epitope was weaker or occurred in a smaller subset of patients than the response to HPV16.

Participants with detectable serum neutralizing activity against HPV16 and HPV31 due to prior exposures confound the analysis of whether neutralizing antibody response to the vaccine could confer protection. When excluding participants with pre-existing HPV16 neutralizing antibodies, passive transfer of sera from patients (*n* = 6) with increased levels of HPV16 neutralizing antibodies at day 71 (D71 L2 16NAb+) exhibited numerically stronger protection against HPV16 challenge at day 71 (not significant) compared to those who did not, and there was no difference at later time points (Fig. 2C). There was no overall group difference (P_gp diff_ = 0.09) and no group difference in change from baseline (P_gp diff in change from D-1_ = 0.29).

When excluding participants with pre-existing HPV31 neutralizing antibodies, passive transfer of sera from patients (*n* = 2) with increased levels of HPV31 neutralizing antibodies at day 71 (D71 L2 31NAb+) exhibited no difference in protection against HPV31 challenge at days 71 or 194 compared to those who did not (D71 L2 31NAb−) (Fig. 2D). This likely reflects the small number of available samples and/or a weak response.

No participants had detectable serum neutralizing activity against HPV5 at baseline (Table 1), and participants who developed an increased HPV5 neutralizing antibody titer at 2 weeks after the second dose were assumed to have developed an L2-specific neutralizing antibody response after vaccination (D71 L2 5NAb+). While there was no overall group difference (P_gp diff_ = 0.64), there was a significant change from baseline at day 194 (*P* = 0.03). Specifically, passive transfer of sera from patients (*n* = 3) with increased levels of HPV5 neutralizing antibodies at day 71 (D71 L2 5NAb+) exhibited significantly stronger protection against HPV5 challenge at day 194, as compared to those who did not, and there was no difference at day 71 (Fig. 2E). This suggests that a subset of the participants developed a cross-protective L2-specific serum antibody response effective against HPV5 after three vaccinations. As for HPV76, no evidence of protection was evident in baseline samples or upon vaccination (Fig. 1E).

### Impact of prior HPV16/31 neutralizing antibodies upon HPV neutralizing antibody response to vaccination

**Fig 3:**
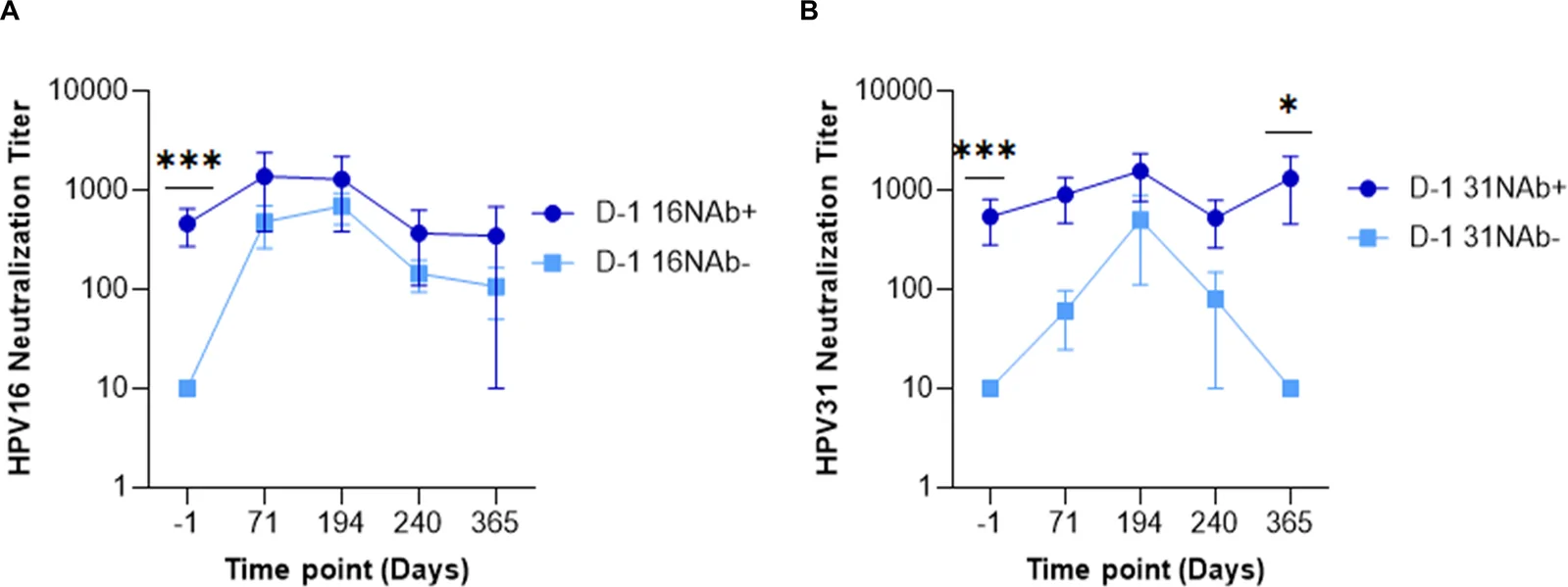
Impact of pre-existing HPV neutralizing antibodies on neutralization response to vaccination. (**A**) Participants in the active arm were stratified based on whether they had neutralizing antibodies to HPV16 at baseline (D-1 16NAb+; *n* = 4) or not (D-1 16NAb−; *n* = 10). Their HPV16 neutralizing response to AAVLP-HPV vaccination was compared. (**B**) Participants in the active arm were stratified based on whether they had neutralizing antibodies to HPV31 at baseline (D-1 31NAb+; *n* = 6) or not (D-1 31NAb−; *n* = 8). Their HPV31 neutralizing response to AAVLP-HPV vaccination was compared. Mean ± SEM. Statistical analysis was performed using unpaired non-parametric Mann–Whitney tests comparing the groups at each time point.

Participants in the active group were stratified based on whether they had detectable HPV16 neutralizing antibodies by fcPBNA at baseline (D-1 16NAb+) or not (D-1 16NAb), and their neutralization titers differed over the study timeline (P_gp diff_ = 0.02). Higher concentrations of HPV16-specific neutralizing antibodies in previously exposed patients suggest a pre-existing level of L1-specific neutralizing antibodies, with a boost in neutralizing titers due to L2-specific antibodies after vaccination, which wane to baseline levels by the end of the study. Neutralization responses against HPV16, compared to baseline, were also significantly different after vaccination (P_gp diff in change from D-1_ = 0.01) (Fig. 3A), indicating greater boosting of HPV16-specific antibodies (i.e., from undetectable) in the HPV16-naïve patients. However, the levels of HPV16 neutralizing antibodies in the HPV16-naïve patients were significantly lower than those of previously exposed patients at baseline and days 71 (P_gp diff_ = 0.03) and 365 (P_gp diff_ = 0.02), but only trended so at days 194 and 240. This implies the HPV16-naïve patients achieved similar titers only after three vaccinations but wane to a greater extent than the previously exposed patients by day 365.

When AAVLP-HPV–vaccinated participants were stratified based on whether they had detectable HPV31 neutralizing antibodies at baseline (D-1 31NAb±), their neutralization titers again differed over the study timeline (P_gp diff_ = 0.002). The greater HPV31-specific neutralizing antibodies in the previously exposed patients suggest a pre-existing level of L1-specific neutralizing antibodies, with a boost in neutralizing titer due to L2-specific antibodies after vaccination. Neutralization responses against HPV31, compared to baseline, were not significantly different after vaccination (P_gp diff in change from D-1_ = 0.41) based on baseline status (Fig. 3B), likely reflecting weak responses in the few HPV31-naïve patients. The levels of HPV31 neutralizing antibodies in HPV31-naïve patients were significantly and consistently lower than for those previously exposed at days −1 (P_gp diff_ < 0.001), 71 (P_gp diff_ = 0.02), 194 (P_gp diff_ = 0.02), 240 (P_gp diff_ = 0.02), and 365 (P_gp diff_ = 0.01). The fact that the HPV31-naïve patients never achieved similar titers even after three vaccinations implies a weaker response to HPV31. Indeed, the HPV31 response to vaccination of HPV31-naïve patients was undetectable by day 365. This was not the case for the HPV16 neutralizing response to vaccination of HPV16-naïve patients, suggesting that the HPV16 L2 epitope is more immunogenic than the HPV31 L2 epitope, or that the display of the latter is in a less favorable position in the AAVLP capsid.

These findings suggest that prior HPV16 or HPV31 exposure had a significant positive impact on overall neutralization responses to AAVLP-HPV vaccination, in part due to the continued presence of a pre-exisiting L1-specific response that added to (but also masked) the L2-specific neutralizing response to AAVLP-HPV vaccination.

### Impact of prior HPV16/31 neutralizing antibodies on L2-specific response to vaccination

To characterize L2-specific responses to AAVLP-HPV vaccination, we measured antibody reactivity to HPV16 and HPV31 L2 peptides by ELISA (Fig. S1A and B). Most participants lacked detectable L2-specific antibodies at baseline, as HPV infection typically induces an L1-specific, but not L2-specific, antibody response. Likewise, only a small subset exhibited titers at day 15—three participants for HPV16 L2 and two for HPV31 L2—suggesting a possible memory response. Notably, the same two individuals showed pre-existing reactivity to both peptides. By 2 weeks after the second vaccination (day 71), approximately half of the participants mounted measurable L2-specific antibody responses that were sustained for the remainder of the study, whereas the other half showed transient titer spikes after each dose that declined rapidly. No L2-specific antibodies were detected in any placebo recipients at baseline or at subsequent time points (Fig. S1A and B).

To assess whether prior HPV16 exposure influenced HPV16 L2-specific responses, participants were stratified by the presence of HPV16 neutralizing antibodies at baseline (D-1 16NAb+) or their absence (D-1 16NAb−). While the HPV16 L2 peptide ELISA titers (Fig. S1C) of the two groups were not significantly different, indicating that prior HPV infection did not substantially affect L2-specific antibody responses after AAVLP-HPV vaccination, they were numerically lower in the HPV16-naïve cohort. In a parallel analysis assessing whether prior HPV31 exposure influenced HPV31 L2-specific responses to vaccination (Fig. S1D), participants with HPV31 neutralizing antibodies at baseline (D-1 31NAb+) exhibited numerically lower HPV31 L2 peptide titers in serum samples after vaccination than those lacking HPV31 neutralizing antibodies at baseline (D-1 31NAb−). Together, these analyses suggest that a prior infection primes the immune system to respond with an enduringly higher L2 antibody response following AAVLP-HPV vaccination. We speculate that prior natural HPV infection primes the immune response to L2 17–36, resulting in strong L2-specific antibody responses to AAVLP-HPV vaccination in previously exposed individuals. By contrast, HPV-naïve participants exhibit comparatively weaker L2-specific antibody responses after AAVLP-HPV vaccination, likely because the AAV epitopes in the vaccine are more numerous and/or dominant compared to the relatively small L2 17–36 peptides.

We next evaluated whether participants who demonstrated a memory-like HPV16 L2 peptide response at day 15 (D15 L2Ab+) exhibited enhanced responses to vaccination. However, no meaningful differences in subsequent HPV16 L2 antibody responses (Fig. S1E) or neutralization titers (Fig. S1F) were observed based on this stratification criterion. A similar trend was noted for HPV31 (Fig. S2A and B).

The same stratification approach was applied to evaluate whether an early L2-specific memory response influenced the protective efficacy of vaccination, which showed no meaningful differences (Fig. S2C). However, interpretation of this analysis is constrained by the very small number of D15 L2Ab+ participants (*n* = 3 for HPV16; *n* = 2 for HPV31), limiting the ability to draw meaningful conclusions about their subsequent responses to infection. Participants who maintained an HPV16 L2-specific antibody response at day 365 (D365 L2Ab+) exhibited protective responses against HPV16 that were comparable to those without sustained L2 antibodies (Fig. S2D). However, a parallel analysis for HPV31 suggested a significant decrease in protection in participants who lacked detectable HPV31 L2 titers at day 194 (Fig. S2E).

### Breadth of L2-specific antibody response

AAVLP-HPV vaccination induced a robust antibody response against HPV16 and HPV31 L2 17–36 peptides in most participants by 2 weeks after the third vaccination (day 194) (Fig. S1A and B). Strongly significant responses were evident against L2 peptides of HPV5/8, 18, 35, 39, 45, 52/58, and 59; less pronounced responses were observed for HPV51 and 56, weaker responses for HPV6, and no significant response was seen for HPV76 or the no-peptide control (Fig. S3).

Participant 13 exhibited the strongest ELISA titers against HPV16 and 31 L2, as well as all other clinically relevant HPV types tested (Fig. S4A), while no evidence of pre-exposure to any hrHPV or HPV6/11 was evident. At 2 weeks after the third immunization, neutralizing titers were detected against HPV6, 16, 31, 33, 35, 45, and 59, as well as against the betapapillomaviruses HPV5 and 92. However, neutralization of HPV11, 18, 51, 52, 58, and 68 was not observed, despite lower but significant reactivity against their L2 17–36 peptide by ELISA. This suggested that some cross-reactive L2 peptide antibody responses were not of sufficient avidity to achieve neutralization, likely due to lower sequence conservation within the L2 17–36 epitope of the more distantly related types. While other participants exhibited strong neutralizing responses similar to subject 13 (e.g., 6 and 11), analysis of their cross-reactive response is confounded by the presence of hrHPV-neutralizing antibodies in their baseline sera.

Subject 13 also demonstrated a strong protective response following vaccination. Their serum conferred protection against HPV16, HPV31, and HPV5, and to a lesser extent, against HPV76 (Fig. S4B through E). Because subject 13 was HPV-naïve at baseline, they served as an example for evaluating vaccine-induced protection without the confounding effects of prior infections. Together, these findings indicate that AAVLP-HPV vaccination is capable of eliciting a cross-reactive L2 antibody and protective response in a hrHPV-naïve individual.

### Assessment of L2-specific antibody avidity by blocking ELISA

A blocking ELISA was developed based upon WW1, a rat monoclonal antibody that binds to HPV16 and HPV31 L2 17–36 peptides with Kd values of 37 nM and 261 nM, respectively (8). WW1 neutralizes HPV16 *in vitro* (IC50 26 nM) but failed to neutralize HPV31. Passive transfer of WW1 protected mice against vaginal challenge with HPV16, but not HPV31 (8). Thus, a human antibody able to compete with the binding of WW1 to HPV16 and HPV31 L2 is likely to exhibit sufficient avidity to be protective, but not if it is only competitive for HPV31 binding by WW1. As a positive control, a rabbit antiserum against KLH-coupled HPV16 L2 17–36 peptide that is both broadly cross-neutralizing and protective by passive transfer was utilized for blocking (12). Briefly, microtiter plates coated with HPV16 or HPV31 L2 peptide were reacted with dilutions of the participant sera (or positive control sera) and then probed with WW1 and anti-rat IgG secondary antibody.

**Fig 4:**
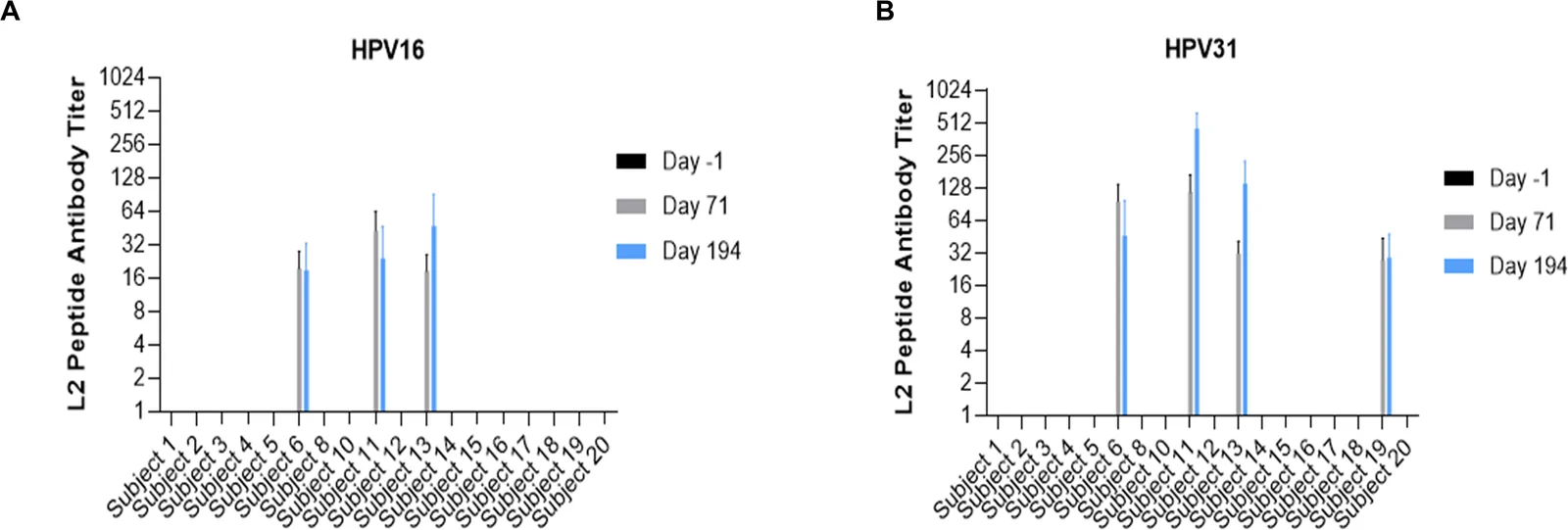
Sera from only a subset of AAVLP-HPV–vaccinated participants exhibited robust avidity for HPV16 and HPV31 L2-peptide. Participants’ sera (or rabbit antiserum against KLH-coupled HPV16 L2 17–36 peptide as a positive control) were diluted 2-fold from 1:25, reacted with plates coated with (**A**) HPV16 or (**B**) HPV31 peptide, and used to compete binding of rat monoclonal antibody WW1. Baseline signal for this assay was defined as a titer of 12.5. Bound WW1 was detected using an anti-rat IgG-HRP secondary antibody. The resulting HRP signal inversely reflects the level of serum antibodies blocking WW1 binding to the target peptide. Titers (mean ± SD) of human sera that are able to compete for 50% of the WW1 binding signal are presented.

A blocking ELISA confirmed these observations, revealing detectable HPV16- and HPV31-specific antibody titers at days 71 and 194 (2 weeks after the first and second boosters). Participant 13 demonstrated a strong response blocking binding of WW1 to both HPV16 and HPV31 L2 peptides (Fig. 4A and B), as did participants 6 and 11. Participant 19 was also naïve to HPV16 and HPV31 at study entry, but AAVLP-HPV vaccination only induced a response capable of blocking WW1 binding to HPV31, but not to HPV16 L2. This suggests a lower avidity response than produced by participant 13. No other participants produced an L2-specific antibody response of sufficient avidity to compete WW1 binding to either HPV16 or HPV31 L2 peptide.

### Antibody response to the vaccine antigen and impact of prior HPV16/31 exposure

To assess whether pre-existing HPV16/31 neutralizing antibodies influenced responses to the vaccine construct, we first measured antibodies specific to the AAVLP-HPV antigen by ELISA. Most participants, including those in the placebo group, showed measurable antibody titers against the AAVLP-HPV antigen at day −1 (Fig. S5A). Given the generally weak or absent L2-specific responses observed in most participants at baseline (Fig. S1A and B), these signals were most likely directed against VP3 of AAV2.

We then stratified participants in the active group based on the presence of baseline HPV16 neutralizing antibodies (D-1 16NAb±) and compared their AAVLP-HPV ELISA responses. Mixed-effects modeling found no significant differences over time between participants with prior HPV16 exposure as compared to naïve participants (Fig. S5B), although the response overall was lower in the latter group. A similar analysis for HPV31 showed no difference (Fig. S5C). Together, these findings suggest that the dominant antibody response after AAVLP-HPV vaccination is against AAV2 VP3 rather than L2.

### Impact of AAVLP-HPV vaccination on VP3-specific antibody responses

The majority of the vaccine antigen comprises the VP3 capsid protein, which displays neutralizing AAV2 epitopes as well as L2 peptides on the VLP surface. We examined the antibody response to vaccination by AAVLP-HPV ELISA (Fig. S5A), and as expected, the AAVLP-HPV antibody titers in the placebo group did not change significantly across each time point. AAVLP-HPV vaccination induced a robust antibody response in all participants. Notably, a dramatic increase in titer was evident in many participants by day 15 (2 weeks after the first dose), with relatively minimal changes thereafter despite two more vaccinations. This implies a robust memory recall response. In the remainder, the antibody titer increased further after additional vaccinations, but tended to wane over time. This pattern is more consistent with vaccination of an AAV2-naïve host.

### Induction of AAV2 neutralizing antibodies by AAVLP-HPV vaccination

**Fig 5:**
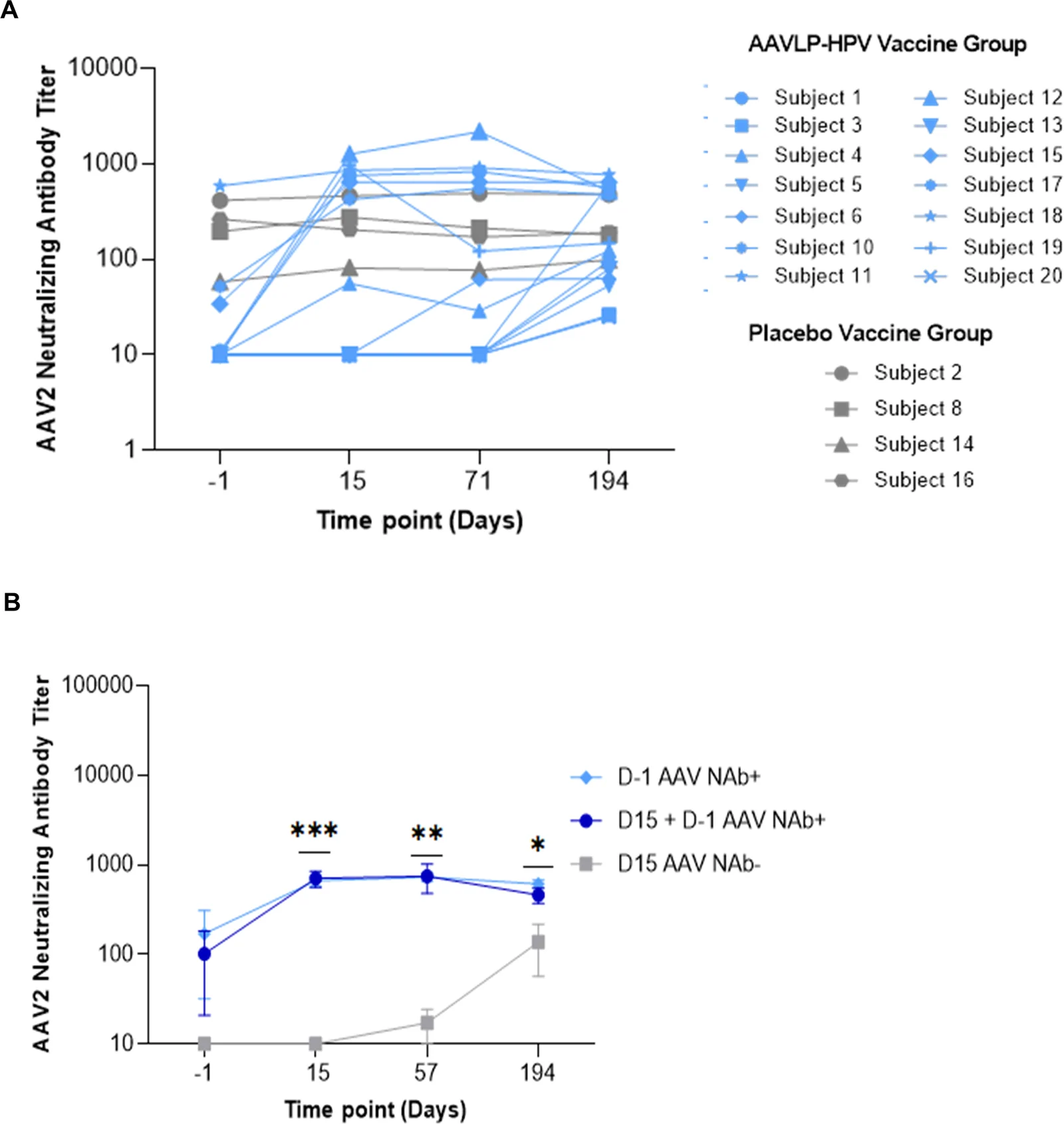
AAV2 neutralizing antibody response in sera of participants vaccinated with AAVLP-HPV or placebo. (**A**) AAV2-Luc PBNA was used at a fixed MOI of 2,000 vge/well and mixed with an equal volume of sera. Titers are reported as logIC₅₀ values based on neutralization curves beginning at a 1:10 dilution of the sera, with a baseline fixed at 10. (**B**) Participants in the active group were stratified based on whether they had AAV NAb at baseline (D-1 AAV NAb+; *n* = 4) or by day 15 (D15 + D-1 AAV NAb+; *n* = 7), and their neutralization responses to AAV2 particles compared to AAV2-naïve participants (D15 AAV NAb−; *n* = 7) were evaluated. Data are presented as mean ± SEM. The *P*-values shown represent comparisons between the D15 + D-1 AAV NAb+ and D15 AAV NAb− groups at each time point using the Wilcoxon rank-sum tests. *P < 0.05, **P < 0.01, ***.

AAV2 infection is common in the general population and typically induces a neutralizing antibody response. To assess a history of AAV2 infection, we implemented a well-characterized AAV2 *in vitro* neutralization assay using pseudovirions carrying a luciferase marker (AAV2-luc) (20). HEK293TT cells were infected at an MOI of 2,000 vg/well of AAV2-luc after incubating with sera diluted 1:10 for all participants; 8/20 had measurable titers (Table 1), ranging from 11 to 586. To understand whether AAVLP-HPV vaccination also induced and/or boosted this response, we assessed the AAV2 neutralizing antibody levels in the serum at the remaining time points (Fig. 5A). Titers in the placebo group did not change over time. Most participants in the active arm demonstrated a robust jump in AAV2 neutralizing antibody titer by day 15, 2 weeks after the first vaccination. This suggests a memory recall response to a prior AAV2 infection. The remainder only developed robust AAV2 neutralizing titers after two vaccinations (day 71), suggesting that VP3 epitopes in the AAVLP-HPV vaccine elicited this response (Fig. 5A and B). After stratifying participants based on whether they had pre-existing AAV2 neutralizing antibodies at baseline (D-1 AAV NAb±) or demonstrated a response at day 15 (D15 AAV NAb±) versus those with neither (D15 AAV Nab−), the latter group exhibited significantly lower titers (P_gp diff_ = 0.01) and a significantly lower response from baseline (P_gp diff in change from D-1_ = 0.003). Post hoc analyses showed that the AAV2-naïve participants exhibited significantly lower AAV2 neutralizing responses at day 57 after the first vaccination and day 194, 2 weeks following the third vaccination, compared to those with prior exposure (Fig. 5B). Overall, this analysis also showed that AAV2-naïve participants required three vaccine doses to achieve significant AAV2 neutralizing titers, whereas a single AAVLP-HPV vaccination of previously AAV2-infected participants was sufficient to produce an even higher titer.

### Impact of prior AAV2 infection on L2-specific responses to vaccination

**Fig 6:**
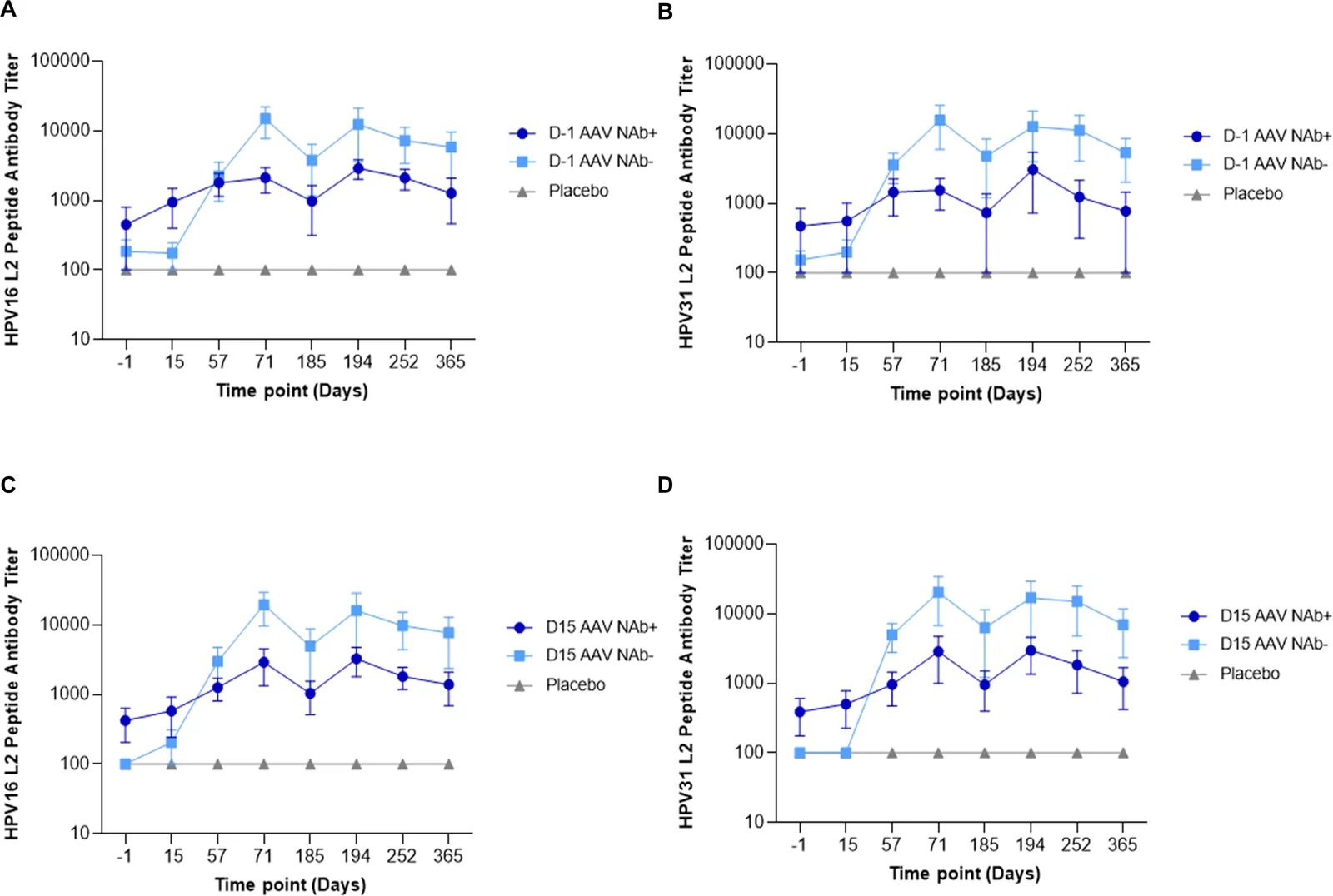
Impact of prior AAV2 infection on L2-specific responses to vaccination. Participants in the active group (*n* = 14) were stratified (**A and B**) based on whether they had AAV NAb at baseline (D-1 AAV NAb+; *n* = 4) or (**C and D**) based on evidence of an AAV memory response (D15 AAV NAb+; *n* = 7), and L2 peptide responses against HPV16 and HPV31 were compared against placebo (*n* = 4). Data are shown as mean ± SEM.

Participants in the active vaccine group were stratified based on the presence of measurable AAV2 neutralizing titers at baseline (D-1 AAV NAb+) or their absence (D-1 AAV NAb−). Participants who lacked AAV2 neutralizing antibodies at day −1 exhibited a robust L2-specific antibody response beginning 56 days after the first AAVLP-HPV vaccination and peaking by 2 weeks after the second dose (day 71) (Fig. 6A and B). The response waned slightly by day 185, returned to peak levels 2 weeks after the third dose, then waned slightly and stabilized at a robust level by day 365. In contrast, participants with detectable AAV2 titers at day −1 exhibited a consistently dampened L2 antibody response even following the full three-dose regimen. This pattern also held when stratifying participants who had detectable AAV2 neutralizing antibodies at day 15 (D15 AAV NAb+) versus those who did not (D15 AAV NAb−) for HPV16 (Fig. 6C) or HPV31 L2 antibody responses (Fig. 6D). Although no robust differences were observed between the stratified groups over time, changes from baseline differed significantly between groups for HPV16 L2 (P_gp diff in change from D-1_ = 0.006) and HPV31 L2 (P_gp diff in change from D-1_ = 0.001). Altogether, these data suggest that prior AAV2 exposure negatively impacted L2-specific responses.

### Impact of prior AAV2 infection on HPV16 and HPV31 neutralizing antibody responses to vaccination

**Fig 7:**
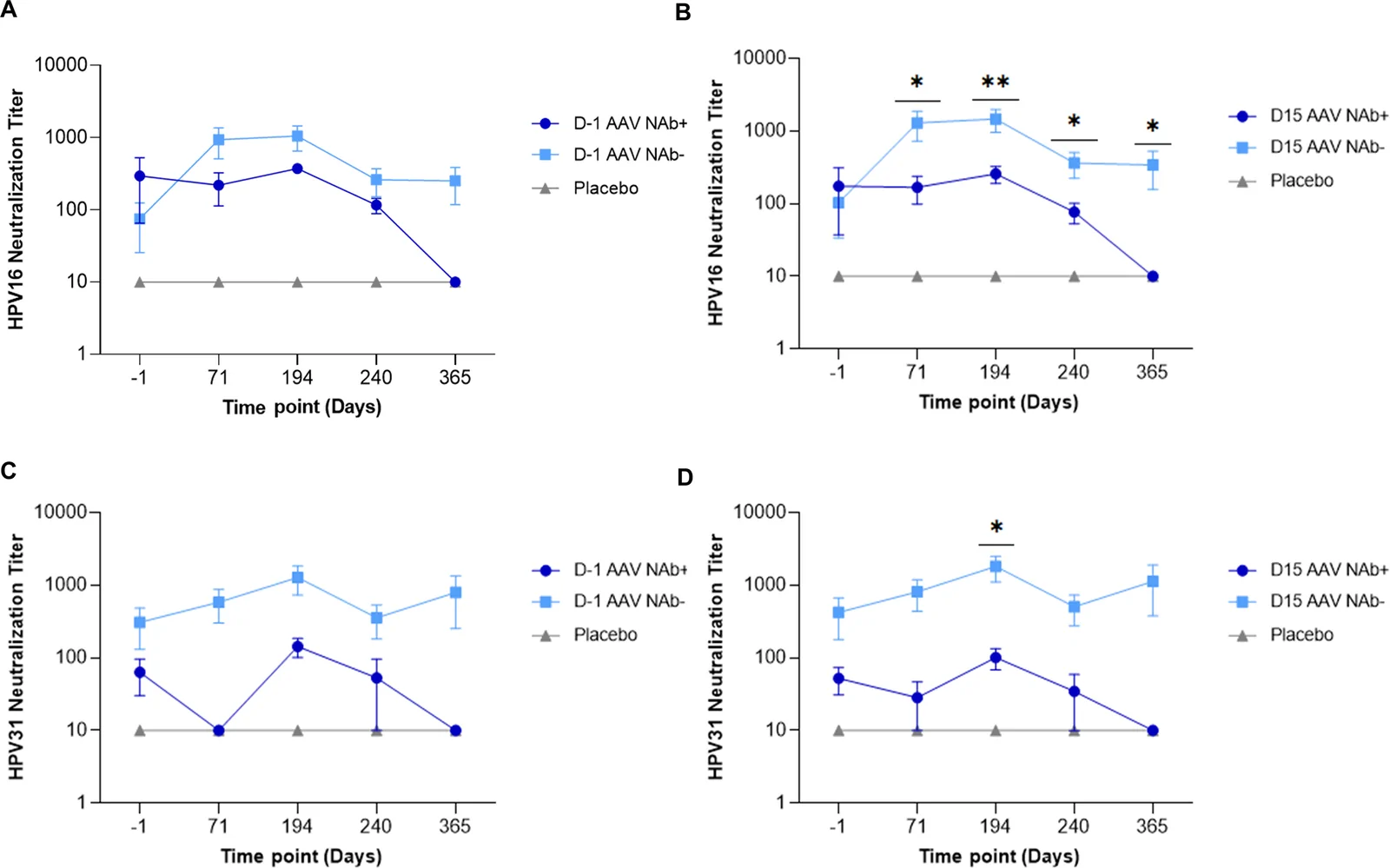
Impact of prior AAV2 infection on HPV neutralizing antibody responses to vaccination. Participants in the active vaccine group (*n* = 14) were stratified by the presence of AAV2 neutralizing antibodies (D-1 AAV NAb+; *n*=4) at baseline (**A**) or by evidence of a memory AAV2 response at day 15 (D15 AAV NAb−; *n* = 7) (**B**). Neutralizing antibody responses to HPV16 (**A, B**) and HPV31 (**C, D**) were then compared between groups. Data are shown as mean ± SEM. The *P*-values shown represent comparisons between the AAV NAb+ and AAV NAb− groups at each time point using the Wilcoxon rank-sum tests, after first assessing overall group differences and differences in change from baseline using a mixed-effects model. *P < 0.05, **P < 0.01.

We then assessed the impact of prior AAV2 exposure on vaccine-induced neutralizing antibody response. HPV16-naïve participants who had no detectable AAV2 neutralizing antibodies at day −1 (*n* = 2) exhibited a significant increase in HPV16 neutralizing antibody titers at day 71, 2 weeks after the second AAVLP-HPV vaccination, with no further increase after the third dose (day 194) (Fig. 7A). The response waned slightly by day 240 and stabilized at a robust level by day 365. In contrast, participants with detectable AAV2 titers at day −1 exhibited no HPV16 neutralizing antibody response, even following the full three-dose regimen, and the titer waned to undetectable levels by day 365. This same pattern was evident when stratifying for participants with detectable AAV2 neutralizing antibodies at day 15 (*n* = 5) (Fig. 7B). Those with prior AAV2 exposure exhibited lower overall levels of HPV16 neutralizing antibodies (P_gp diff_ = 0.02) and a trend for a weaker response from baseline (P_gp diff in change from D-1_ = 0.07). A parallel analysis for HPV31 neutralizing antibody responses was performed (Fig. 7C and D). Those with prior AAV2 exposure exhibited lower overall levels of HPV31 neutralizing antibodies (P_gp diff_ = 0.047) but no significant difference in the response from baseline (P_gp diff in change from D-1_ = 0.29). However, in participants who lacked AAV2 neutralizing antibodies at day −1 (or day 15), the increase in HPV31 neutralizing antibody titer was less evident than that observed for HPV16. This likely reflects the higher baseline level of HPV31 neutralizing antibodies in this group and/or less effective presentation of the HPV31 L2 epitope compared to HPV16. In addition, the HPV31 L2 peptide-specific responses were also overall weaker than those seen for the HPV16 L2 peptide, raising the possibility that the latter peptide is more immunogenic per se or is displayed at a more immunogenic site within the VP3 capsid.

### Impact of prior AAV2 infection on protective antibody responses against HPV types 16, 31, 5, and 76

**Fig 8:**
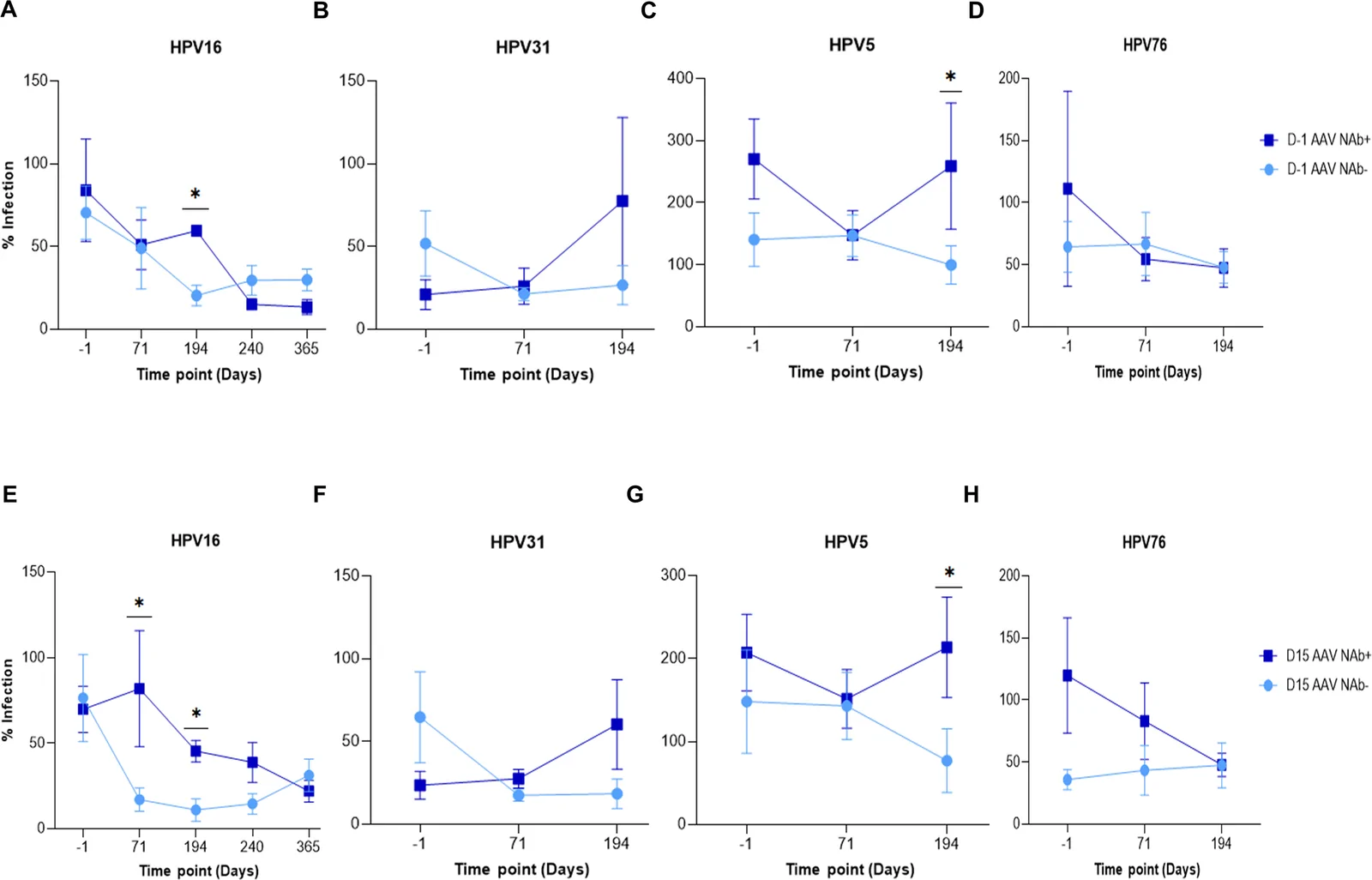
Impact of prior AAV neutralizing antibodies on protective responses by participants vaccinated with AAVLP-HPV. After excluding participants with pre-existing neutralizing antibodies at baseline, subjects in the AAVLP-HPV vaccine group (*n* = 14) were stratified according to their AAV2 NAb response on (**A–D**) day −1 (*n* = 4 for D-1 AAV NAb+ and *n* = 10 for D-1 AAV NAb−) or (**E–H**) day 15 (*n* = 7 for D15 AAV NAb+ and *n* = 7 for D15 AAV NAb−) and analyzed for their protective response against HPV16 (**A and E**), HPV31 (**B and F**), HPV5 (**C and G**), or HPV76 (**D and H**). The *P*-values shown represent comparisons between the AAV NAb+ and AAV NAb− groups at each time point using the Wilcoxon rank-sum tests, after first assessing overall group differences and differences in change from baseline using a mixed-effects model. *P < 0.05, **P < 0.01.

Lastly, we analyzed the response to AAVLP-HPV vaccination by passive transfer of sera from participants into mice and vaginal challenge with HPV16 and compared the level of protection conferred by longitudinal serum samples from those with or without AAV2 neutralizing antibodies at day −1 (D-1 AAV NAb±). Protection was evident 2 weeks after the third vaccination of participants without AAV2 neutralizing antibody at baseline, and the protection persisted through day 365 (Fig. 8A). Interestingly, significant protection against HPV16 compared to baseline only emerged at day 240, and it remained at day 365. When stratifying for the participants who had no AAV2 neutralizing antibodies at day 15 (D15 AAV NAb−), significant protection against HPV16 was evident 2 weeks after the second immunization (day 71) and persisted (Fig. 8E). The protective response compared to baseline was delayed until day 240 in participants who were AAV2 neutralizing antibody positive at day 15 (D15 AAV NAb+).

Many participants had robust titers of HPV31 neutralizing antibodies regardless of AAV2 serostatus (Table 1), presumably from prior natural exposure, which conferred protection against HPV31 challenge. As a result, the potency of protection against HPV31 conferred by passive transfer did not differ significantly between groups with or without AAV neutralizing antibodies present at day −1 (Fig. 8C); the same was observed between groups with or without AAV neutralizing antibodies present at day 15 (P_gp diff_ = 0.10) (Fig. 8F). For those with no AAV2 neutralizing antibody titer at day 15, there was a trend toward greater potency of protection against HPV31 conferred by passive transfer (P_gp diff in change from D-1_ = 0.08) (Fig. 8F).

None of the participants had HPV5 neutralizing antibodies at study entry. Interestingly, a protective response against HPV5 emerged 2 weeks after the third AAVLP-HPV vaccine in the participants lacking AAV2 neutralizing antibodies at day −1 (Fig. 8C), but not in those with baseline AAV2 neutralizing titers. This protective response was also evident for those lacking AAV2 neutralizing antibodies at day 15 (i.e., no AAV2 immune memory) (Fig. 8G). In contrast, no protection was seen for HPV76 (Fig. 8D and H), which likely reflects the greater sequence similarity of HPV5 L2 17–36 sequence to that of HPV16 compared to the more divergent HPV76.

## DISCUSSION

The use of adjuvants with prophylactic vaccines is well understood to enhance the immune response (21). However, potential concerns about the safety of adjuvants in healthy patients suggest the value of demonstrating the necessity of their inclusion. In this study, participants received three 20 µg doses of AAVLP-HPV without the benefit of an adjuvant. L2-specific antibody responses were evident in the vaccinated patients, but were weak and short-lived in a subset. Only a few patients produced high avidity antibodies against HPV16 L2. HPV16 and HPV31 neutralizing antibody responses to AAVLP-HPV vaccination were detected in some patients but were weak overall. Nevertheless, passive transfer of the immune sera from some patients conferred protection against HPV16 and HPV31 and even cross-protection against HPV5. Preclinical studies suggested that active AAVLP-HPV vaccination without an adjuvant was protective, but neutralizing antibody responses were only detectable (titer >50) when an adjuvant was used. Cross-protective responses against HPV76, whose L2 17–36 sequences are more divergent, were not evident. Importantly, the protection achieved by passive transfer of 100 µL patient serum (~1:20 dilution in a mouse) conferred only partial protection (≤90%) against HPV16 or HPV31 challenge, as opposed to the potent (≤99%) protection achieved using serum of rabbits vaccinated with Gardasil9. These observations suggest the need for an adjuvant to achieve robust protection by AAVLP-HPV vaccination (16, 17, 21).

While preclinical studies of AAVLP-HPV vaccination in laboratory mice and rabbits elicited a clear L2-specific antibody response associated with protection against HPV challenge (17), interpretation of the finding in this phase I study has been challenged by prior infectious exposures in the participants. First, most patients had evidence of prior natural exposure to various hrHPV, resulting in detectable levels of neutralizing serum antibodies. Such responses in baseline sera of multiple participants were sufficient to confer protection against experimental challenge with HPV16 or HPV31 via passive transfer. These neutralizing responses are typically directed against L1 (22, 23), and indeed, only two patients had HPV16 or HPV31 L2-specific antibodies detectable by ELISA at baseline. Given the small number of patients in the study, these pre-existing L1-specific neutralizing antibody responses clouded interpretation of the protective immunity elicited by vaccination.

Prior natural exposure to HPV16 or HPV31 had a clear impact on the magnitude of the L2-specific antibody response and on the overall increase in neutralizing antibody titers following vaccination. Neutralization responses to the vaccine were higher in subjects with detectable HPV16 neutralizing antibodies at baseline. Three vaccine doses were required to achieve robust HPV31 neutralizing titers in naïve individuals but only two for HPV16. These findings suggest that the vaccine-induced response to HPV31 was generally less robust than that to HPV16. Prior exposure to HPV16 appeared to enhance protective responses (Fig. 2).

A prior infection with AAV2 had a profound effect on responses to AAVLP-HPV vaccination. Participants with AAV2 neutralizing antibodies at baseline, or with an AAV2 memory response (evident based upon dramatically elevated neutralizing titers at day 15), produced only weak, delayed, and short-lived antibody responses to AAVLP-HPV vaccination. By contrast, they produced a robust and enduring AAV2 neutralizing antibody response after only one dose, providing evidence of original antigenic sin (24, 25). Those participants naïve to AAV2 did develop AAV2 neutralizing antibody responses, but only after two or three vaccinations, and these responses were weaker than those seen in participants with a prior AAV2 exposure.

In earlier preclinical studies, mice vaccinated twice with wild-type AAV2 particles generated robust levels of AAV2 capsid antibodies by 1 month later (16). When these mice were subsequently vaccinated with AAVLP-HPV formulated in Montanide adjuvant, they generated an L2-specific antibody response similar to that of mice that had not received prior vaccination with wild-type AAV2 particles. This suggests that the use of an adjuvant with the AAVLP-HPV vaccine might overcome the suppression of the L2-specific response seen in patients with prior exposure to AAV2 by enhancing stimulation of the innate immune system (16, 25). Indeed, without adjuvant, AAVLP-HPV vaccination elicited a very weak serum antibody response in only a subset of mice and only at a higher dose (6 µg versus 0.6 µg per dose) (17). This antibody response failed to detectably neutralize HPV16 (titer ≥ 50). Mice vaccinated at 0.6 µg per dose were not protected against HPV16 challenge, and those receiving 6 µg AAVLP-HPV per dose were only partially protected at 2 weeks after the third vaccination. Interestingly, by 19 weeks after the third 6 µg dose vaccination, the mice were protected from HPV16 challenge, but those vaccinated with 0.6 µg AAVLP-HPV were not (17). This weak and delayed protective response resembles that seen in AAV2-naïve patients vaccinated with 20 µg AAVLP-HPV in the absence of adjuvant. Rabbits vaccinated three times with 20 µg AAVLP-HPV (same as human dose) were protected against cutaneous challenge with HPV16/18/31/35/39/58/59 at 6 months post-vaccination even without the use of adjuvant. Similar protection at 12 months after vaccination was seen in rabbits vaccinated with 20 µg AAVLP-HPV per dose formulated with alum + MPL, but protection was not tested without adjuvant (17).

Overall, this small study suggests that the AAVLP-HPV vaccine will require co-formulation with an acceptable adjuvant. Preclinical studies support its use with alum at a minimum, but a more robust adjuvant system would likely be beneficial, e.g., alum + MPL (17). The preclinical studies also support co-formulation of AAVLP-HPV with Cervarix (which contains alum + MPL) as an approach to broaden coverage to all hrHPV (17) and likely many other types associated with human disease. Montanide, although a potent adjuvant and effective with AAVLP-HPV, is likely too reactogenic for this application (16).

## MATERIALS AND METHODS

### Plasmids

The HPV L1 + L2 expression and luciferase/GFP plasmids were from Addgene.

### Enzyme-linked immunosorbent assays

All ELISAs were performed by coating 96-well plates (Thermo Fisher Scientific, 442404) with 200 ng of neutravidin per well. The following day, an RG-1 peptide corresponding to L2 amino acids 17–36 of the indicated genotypes was used at 25 ng per well and incubated overnight at 4°C. The following day, peptide-coated plates were blocked with blocking buffer (4% dry skim milk/1× DPBS/0.02% Tween-20) for 1.5 h at 4°C. Serum samples collected from vaccinated mice or human sera were diluted starting at 1:50, unless otherwise specified, in blocking buffer and serially diluted 3-fold eight times. Plates were incubated in a shaker at 300 rpm for 1 h at room temperature (RT). ELISA plates were washed three times with Wash Buffer (0.01% [vol/vol] Tween-20 in PBS). Following this, 100 μL of anti-mouse IgG-HRP (Sigma, A8667) per well was added at 1:5,000 in blocking buffer and incubated in a shaker at 300 rpm for 1 h at RT. This was followed by three additional washing steps, and 100 μL of TMB substrate (SeraCare, 5120-0047) was added to each well and incubated for 30 min in the dark at RT. This reaction was terminated using 100 μL of Stop Solution (18% [vol/vol] 2N H_2_SO_4_ in ultrapure water). To test antibody isotypes in mouse sera, a mouse typer isotyping kit was used as per the manufacturer’s instructions (Bio-Rad, #172-2055). To analyze the HRP signal, plates were loaded onto a microplate reader (Bio-Rad, Hercules, CA), and final absorbance values were obtained from subtracting absorbances measured at 620 nm from 450 nm. IC50 values were calculated based on the absorbance curves. Each sample was assayed in triplicates, and the average absorbance values were used for further analysis.

The blocking ELISA was done similarly; HPV 16 or 31 peptide-coated 96-well plates were incubated with subject sera diluted 1:25. Negative control rabbit serum specific for L2 69–81 regions (S580-2, 1/29/04, L2 69–81) and positive control rabbit serum specific for L2 17–36 regions (1075-2, 1/3/04, L2 17–36) were diluted 1:500. Human monoclonal rat IgG2a antibody, WW1 (ENVIGO, 1.53 μg/μL), was diluted in blocking buffer accordingly. For the HPV16 peptide, the optimal signal by WW1 was observed at a 1:3,200 dilution and 1:800 for the HPV31 peptide. After washing, 100 μL of diluted WW1 was added and incubated in a shaker at 300 rpm for 1.25 h. HRP-conjugated rabbit anti-rat IgG (Southern Biotech, cat no. 6185-05) was diluted in blocking buffer at 1:5,000. HRP signal was analyzed as described above.

### Pseudovirus preparation

HPV pseudoviruses (PsVs) were produced in HEK 293TT cells (ATCC) by co-transfecting them with the L1L2 expression plasmid and a firefly luciferase-expressing plasmid, which served as a reporter for infection measurement. On the day of transfection, plasmid DNA was mixed with Mirus transfection reagent (TransIT-2020, cat no. MIR5406) in Opti-MEM reduced-serum medium (Gibco, cat no. 31985-070). This transfection mixture was then added to the cells and incubated at 37°C in a humidified 5% CO₂ atmosphere for 44 to 48 h.

Following incubation, the cells were harvested, cell pellets were lysed in an equal volume of lysis buffer (PBS-Mg supplemented with 0.5% Brij 58 and 0.2% Benzonase nuclease), and incubated at 37°C in siliconized tubes for 24 h. After lysis, the NaCl concentration of the lysates was adjusted to 0.8 M NaCl to stabilize the PsV particles. The lysates were then loaded onto a 27%/33%/39% OptiPrep step gradient and separated by ultracentrifugation for 16 h at 40,000 × *g* in an SW40 rotor. Fractions were collected from the interface between the 39% and 33% Optiprep layers.

### *In vitro* neutralization of AAV

To measure anti-AAV2 neutralizing antibodies in the sera from participants enrolled in the phase I clinical trial (NCT03929172), an AAV neutralizing assay was followed as described by Krotova and Aslanidi (20). In short, HEK293TT cells were plated at 20,000 cells per well in a 96-well plate and incubated overnight. Once the cells reached 70% confluence, sera were diluted 1:10 and 2-fold thereafter and mixed with equal volumes of AAV2-luciferase VLPs (VVC-U of Iowa-1983, Iowa City, IA) at a concentration of 1 × 10^10^ vg/mL and incubated for 1 h at 37°C. A total of 10 μL of the AAV/sample mix was added to the cells and incubated for 24 hours. Luciferase activity was measured as described above.

### Measurement of HPV neutralizing antibodies in sera

Serum samples were also used to test (non-GCLP) neutralizing antibody titers against the indicated HPV types using a high-throughput pseudovirion neutralization assay (HT-PBNA) as previously described (26) or the modified furin pre-cleaved assay (27), HT-fc-PBNA (28). The samples were diluted down serially, and the half-maximal inhibitory concentration (IC50) of the neutralizing antibody titers was calculated using the GraphPad Prism 7 software (26).

### Mouse passive transfer and vaginal challenge

CD-1 IGS female mice 16–18 g (Charles River Laboratories) received a subcutaneous injection of 3 mg of medroxyprogesterone (Depo-Provera, Pfizer, New York, NY) 4 days before vaginal challenge to synchronize their estrous cycles. Passive transfer of 100 μL of human serum was used to assess the protective responses of AAVLP-HPV- or placebo-vaccinated participants. The following day, mice were intravaginally challenged with HPV16 PsVs suspended in 20 μL of 3% carboxymethyl cellulose (CMC). Seventy-two hours post-challenge, the mice were anesthetized with isoflurane, and 20 μL of luciferin substrate (7.8 mg/mL, Promega, Madison, WI) was administered intravaginally before imaging. Bioluminescence was captured for 5 min using a Xenogen IVIS Spectrum (Caliper Life Sciences, Hopkinton, MA) imager, and data analysis was performed using Living Image 2.0 software. Luminescence data were quantified as the ratio of radiance signal (in photons per second per square centimeter) in the vaginal region to that in the thoracic region. Imaging analysis and quantification of luminescence in regions of interest were carried out using Living Image 2.5 software (PerkinElmer).

### Statistical analyses

Group means ± standard error of the mean over time were plotted. Titer measurements were log10-transformed for analysis. To evaluate overall group differences and whether temporal changes from baseline differed by group, we fitted mixed-effects models to account for within-participant correlation. Models treated time and group as categorical factors, included their interaction, and included a random intercept. Hypothesis testing was performed hierarchically: an overall group difference and group differences in change from baseline were assessed first via mixed-effects model. If either test reached significance, post hoc comparisons were performed further to compare group differences at individual time points or between-group differences in change from baseline at each follow-up. In addition, overall time differences for assessing serum protection profiles were also examined in the above mixed-effect models; if the overall time difference was significant, post hoc pairwise comparisons of changes in protection profiles over time were performed within the vaccinated and placebo groups. Post hoc *P*-values were corrected for multiple comparisons using the Benjamini-Hochberg procedure (19). Analyses were conducted in GraphPad Prism or R v4.4.3, with all figures generated in GraphPad Prism.

## Data Availability

De-identified individual participant data and the study protocol may be made available to qualified researchers for non-commercial, academic research. Due to commercial sensitivity and intellectual property restrictions, data requests will be reviewed by a joint steering committee. Requests must be sent to 2A Pharma AB (https://2apharma.com/contact) with a formal research proposal. Approved requestors will be required to sign a strict Data Use Agreement (DUA) to protect participant confidentiality, commercial confidentiality, and proprietary rights. Numerical data for all biological experiments associated with the individual figures are available from the corresponding author upon request.

## SUPPLEMENTAL MATERIAL

Figure S1 jvi.00448-26-s0001.tif L2 peptide antibody responses upon AAVLP-HPV vaccination.

Figure S2 jvi.00448-26-s0002.tif Impact of L2 peptide antibody memory on responses to AAVLP-HPV vaccination.

Figure S3 jvi.00448-26-s0003.pdf Comparison of antibody reactivity to L2 peptide of different HPV types by ELISA comparing participants that AAVLP-HPV vaccination.

Figure S4 jvi.00448-26-s0004.tif Broadly reactive and protective response to AAVLP-HPV vaccination by HPV naïve participant.

Figure S5 jvi.00448-26-s0005.tif AAVLP-HPV antibody response in sera of participants vaccinated with AAVLP-HPV or placebo.

Supplemental legends jvi.00448-26-s0006.docx Legends for Fig. S1 to S5.

ASM does not own the copyrights to Supplemental Material that may be linked to, or accessed through, an article. The authors have granted ASM a non-exclusive, world-wide license to publish the Supplemental Material files. Please contact the corresponding author directly for reuse.
