# Supplementary material for: Factors impacting protective immunity conferred by vaccination with adeno-associated virus-like particles displaying human papillomavirus L2 residues 17–36
Source: J Virol. 2026 Aug 21;100(9):e00448-26. doi: 10.1128/jvi.00448-26 (PMC13595945; doi:10.1128/jvi.00448-26)
Supplement: Supplementary file 1 [file jvi.00448-26-s0003.pdf]

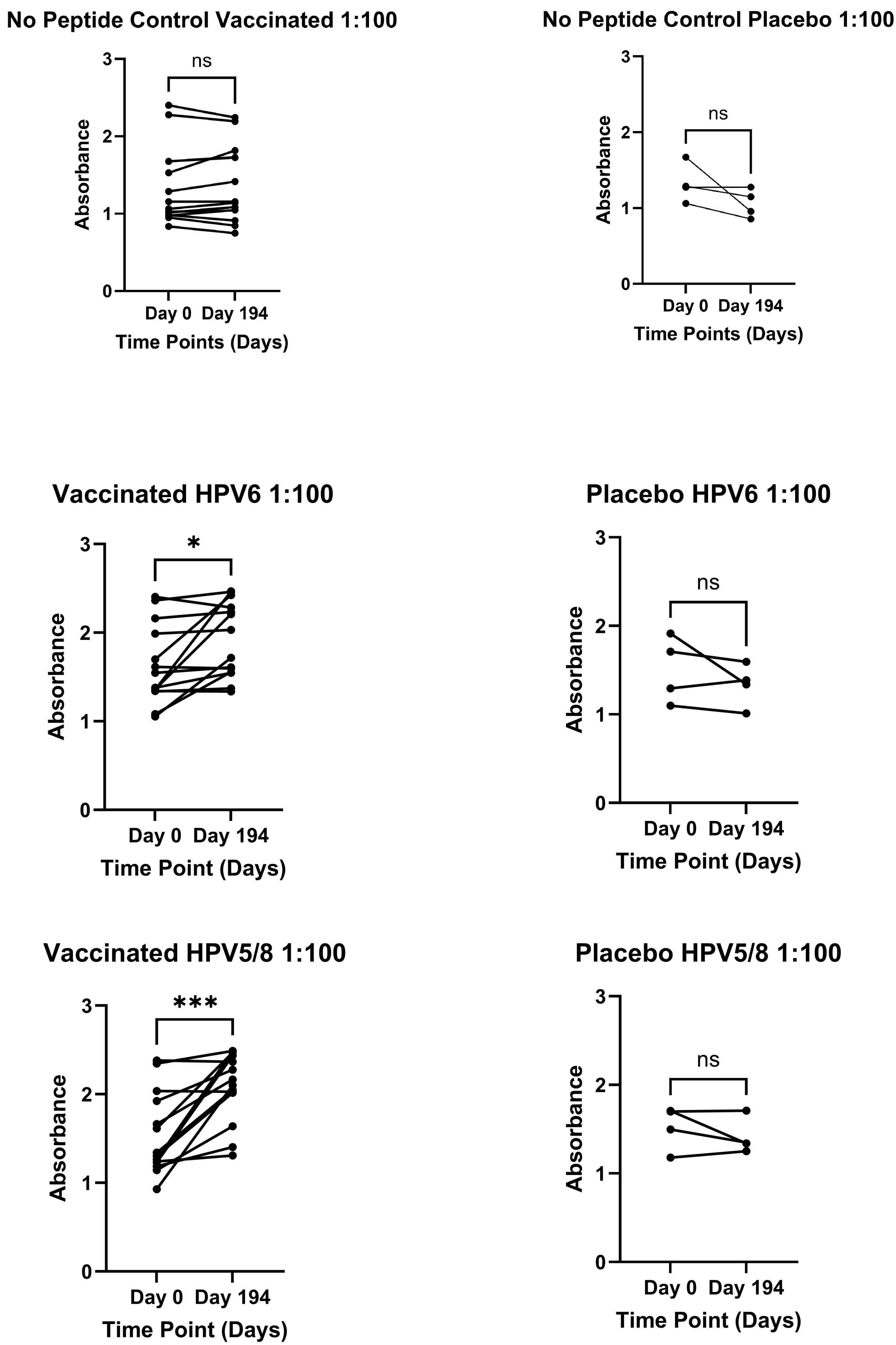
No Peptide Control Vaccinated 1:100
ns
Absorbance
Day 0
Day 194
Time Points (Days)
No Peptide Control Placebo 1:100
ns
Absorbance
Day 0
Day 194
Time Points (Days)
Vaccinated HPV6 1:100
*
Absorbance
Day 0
Day 194
Time Point (Days)
Placebo HPV6 1:100
ns
Absorbance
Day 0
Day 194
Time Point (Days)
Vaccinated HPV5/8 1:100
***
Absorbance
Day 0
Day 194
Time Point (Days)
Placebo HPV5/8 1:100
ns
Absorbance
Day 0
Day 194
Time Point (Days)

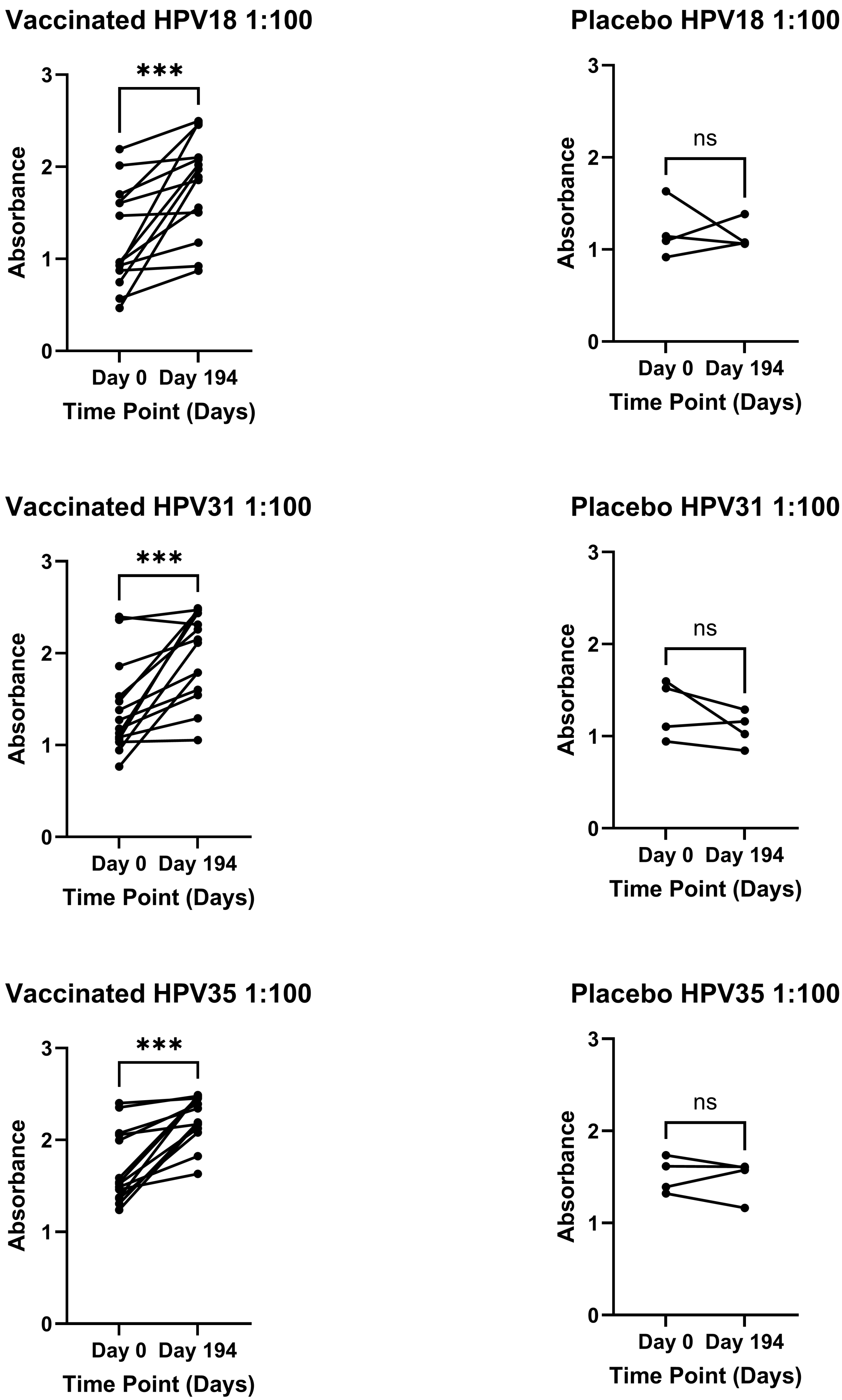
Vaccinated HPV18 1:100
***
Absorbance
0
1
2
3
Day 0
Day 194
Time Point (Days)
Placebo HPV18 1:100
ns
Absorbance
0
1
2
3
Day 0
Day 194
Time Point (Days)
Vaccinated HPV31 1:100
***
Absorbance
0
1
2
3
Day 0
Day 194
Time Point (Days)
Placebo HPV31 1:100
ns
Absorbance
0
1
2
3
Day 0
Day 194
Time Point (Days)
Vaccinated HPV35 1:100
***
Absorbance
0
1
2
3
Day 0
Day 194
Time Point (Days)
Placebo HPV35 1:100
ns
Absorbance
0
1
2
3
Day 0
Day 194
Time Point (Days)

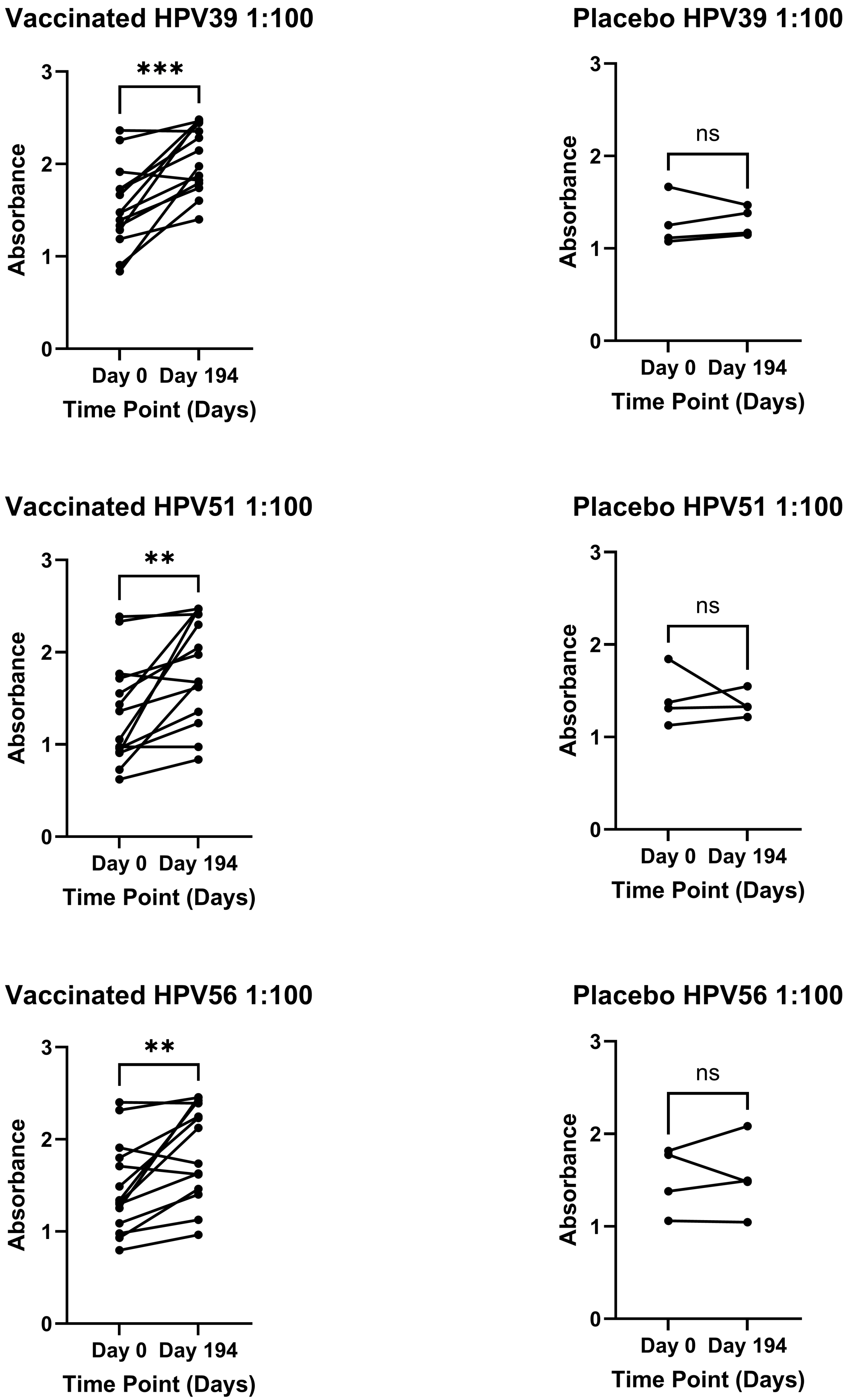

Vaccinated HPV39 1:100
***
Absorbance
Day 0
Day 194
Time Point (Days)
Placebo HPV39 1:100
ns
Vaccinated HPV51 1:100
**
Placebo HPV51 1:100
ns
Vaccinated HPV56 1:100
**
Placebo HPV56 1:100
ns

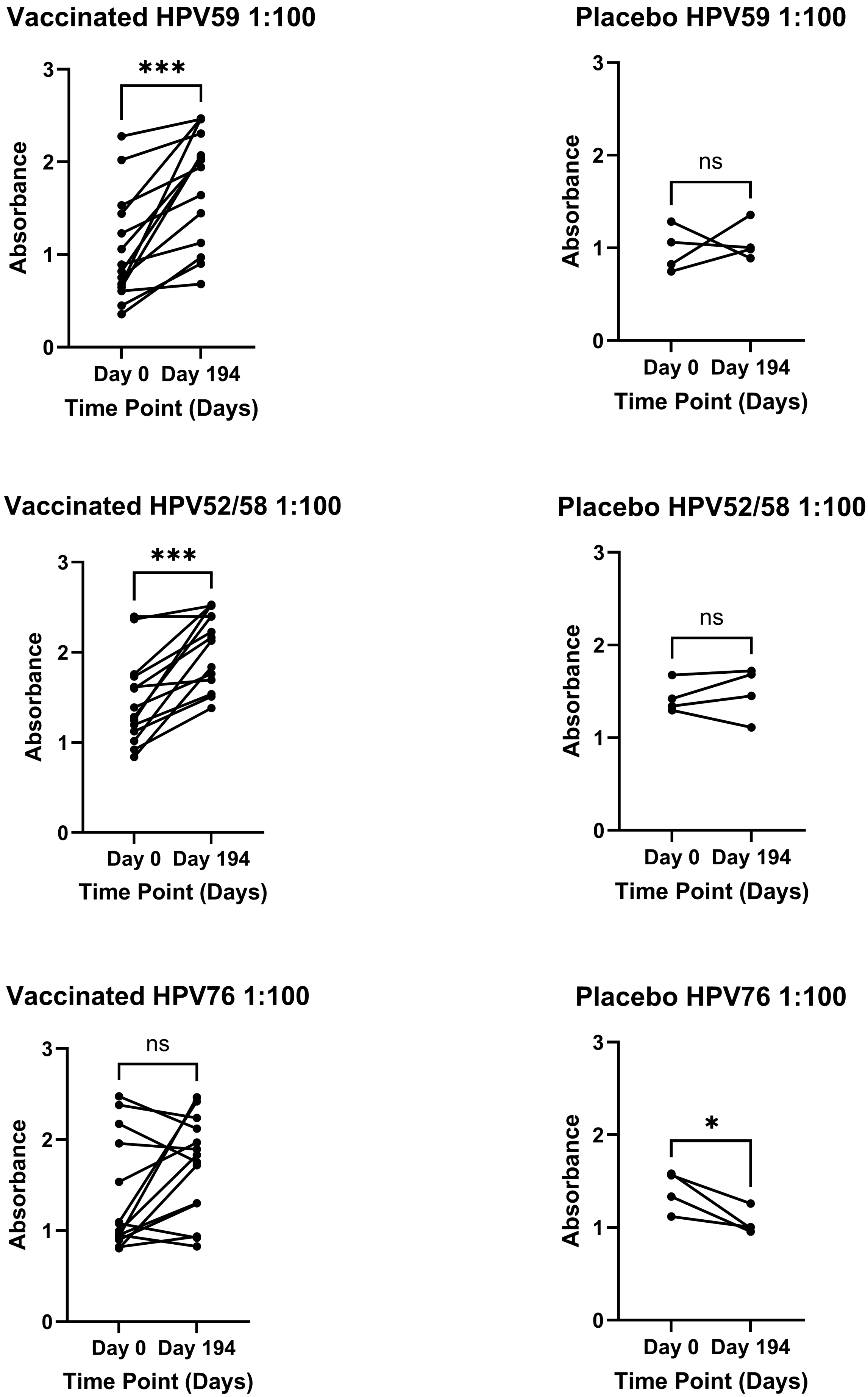
Vaccinated HPV59 1:100
***
Absorbance
0
1
2
3
Day 0
Day 194
Time Point (Days)
Placebo HPV59 1:100
ns
Absorbance
0
1
2
3
Day 0
Day 194
Time Point (Days)
Vaccinated HPV52/58 1:100
***
Absorbance
0
1
2
3
Day 0
Day 194
Time Point (Days)
Placebo HPV52/58 1:100
ns
Absorbance
0
1
2
3
Day 0
Day 194
Time Point (Days)
Vaccinated HPV76 1:100
ns
Absorbance
0
1
2
3
Day 0
Day 194
Time Point (Days)
Placebo HPV76 1:100
*
Absorbance
0
1
2
3
Day 0
Day 194
Time Point (Days)

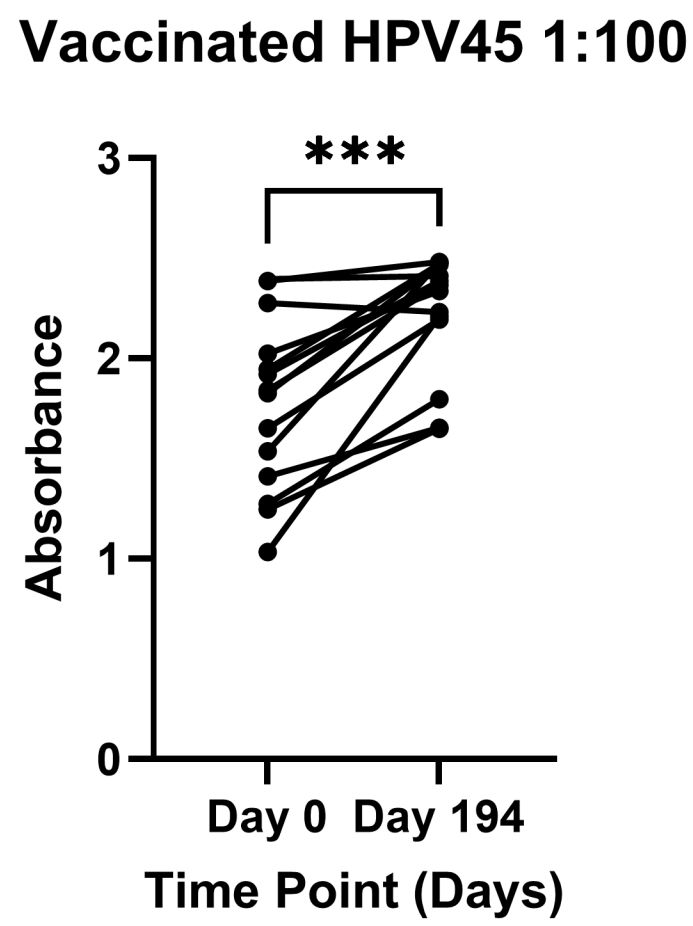
Vaccinated HPV45 1:100
***
Absorbance
3
2
1
0
Day 0
Day 194
Time Point (Days)

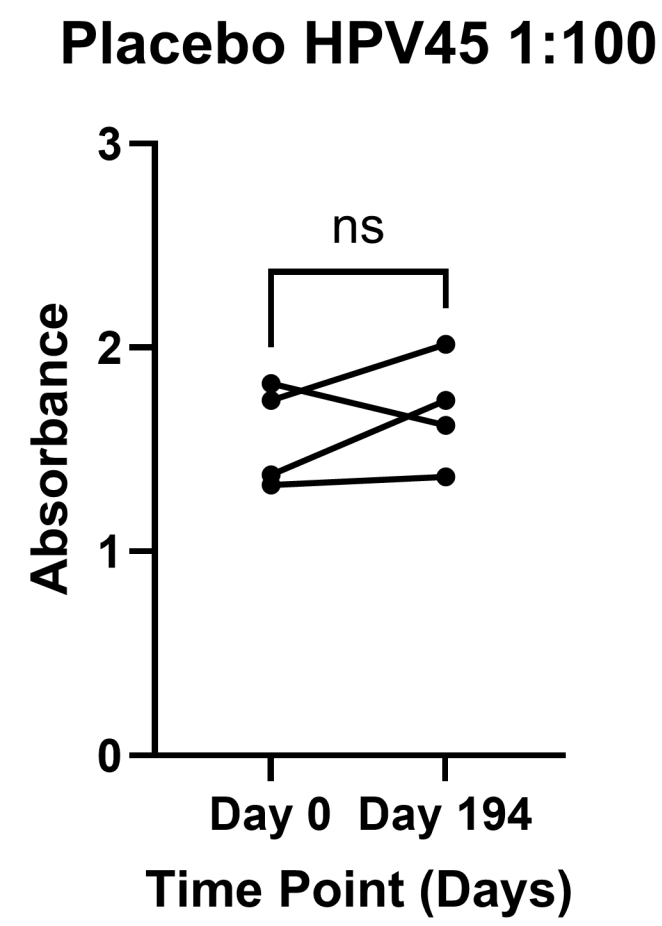
Placebo HPV45 1:100
ns
Absorbance
3
2
1
0
Day 0
Day 194
Time Point (Days)
