# Supplementary material for: Factors impacting protective immunity conferred by vaccination with adeno-associated virus-like particles displaying human papillomavirus L2 residues 17–36
Source: J Virol. 2026 Aug 21;100(9):e00448-26. doi: 10.1128/jvi.00448-26 (PMC13595945; doi:10.1128/jvi.00448-26)
Supplement: Supplementary file 2 [file jvi.00448-26-s0006.docx]

**Supplementary Figure S1: L2 peptide antibody responses upon AAVLP-HPV vaccination.** Serum antibody titers to **(A)** HPV16 L2 and **(B)** HPV31L2 peptide in AAVLP-HPV or placebo vaccinated participants across the study timeline as measured by ELISA. IC₅₀ values are reported as the reciprocal serum dilution required to achieve 50% maximal binding. All participants in the active group were stratified based on measurable HPV neutralizing antibodies at baseline (D-1 NAb+) and their L2 peptide responses to **(C)** HPV16 (n=4 for D-1 16NAb+ and n=10 for D-1 16NAb-) and **(D)** HPV31 (n=6 for D-1 31NAb+ and n=8 for D-1 31NAb-) were compared. Participants were then stratified based on whether they had L2 peptide antibodies by day 15 reflecting either a pre-existing or memory response (n=4 for D15 L2Ab+ and n=10 for D15 L2Ab-). The effect of pre‑existing HPV16 L2 peptide antibodies on the subsequent **(E)** L2 peptide response to AAVLP‑HPV vaccination and **(F)** neutralization response was analyzed. Placebos showed no significant titer throughout the study. Mean ± SEM are shown.

**Supplementary Figure S2: Impact of L2 peptide antibody memory on responses to AAVLP-HPV vaccination.** Participants in the active groups (n=14) were stratified based on whether they had a detectable HPV31 L2 peptide antibodies at day 15 (D15 L2Ab+; n=2) or not (D15 L2Ab-; n=12), as measured by ELISA. **(A)** HPV31 L2 peptide antibody and **(B)** HPV31 neutralizing titers were compared. **(C)** Protection against HPV16 vaginal challenge in mice receiving participant sera was compared between participats who had HPV16 L2 peptide antibodies at day 15 (n=3 for D15 L2 Ab+ and n=11 for D15 L2 Ab-). To assess the role of long‑term antibody response, participants were further stratified by whether they maintained HPV16 and HPV31 antibody titers at day 365, and protection conferred by their sera against **(D)** HPV16 and **(E)** HPV31 challenge was evaluated. Sample sizes were n=7 for HPV16 D365 L2 Ab+ and n=7 for HPV31 D365 L2 Ab+. Mean and SEM shown.

**Supplementary Figure S3: Comparison of antibody reactivity to L2 peptide of different HPV types by ELISA comparing participants that AAVLP-HPV vaccination.** Participants were stratified based on whether they had received AAVLP-HPV vaccination (n=14) or placebo (n=4). ELISA reactivity (absorbance) with L2 17-36 peptide of the indicated HPV types antibody prior to vaccination (Day 0/Day -1) or 2 weeks after the third vaccination (Day 194). Paired t tests were used to compare reactivity between the two time points.

**Supplementary Figure S4: Broadly reactive and protective response to AAVLP-HPV vaccination by HPV naïve participant. (A)** Subject 13 sera were tested beginning at a 1:100 dilution and subsequently diluted 3‑fold. LogIC₅₀ values were calculated from absorbance curves generated starting at the 1:100 dilution. Representative luminescence images of mice (n=3 per timepoint) challenged with **(B)** HPV16, **(C)** HPV31, **(D)** HPV5, and **(E)** HPV76 after passive transfer of subject 13 sera obtained at baseline and at the indicated timepoints after vaccination. Percent infection relative to pre-immune sera (Day -1) is indicated.

**Supplementary Figure S5: AAVLP-HPV antibody response in sera of participants vaccinated with AAVLP-HPV or placebo. (A)** Serum antibody responses to AAVLP-HPV antigen in AAVLP-HPV or placebo vaccinated participants across the study timeline as measured by ELISA. IC₅₀ values are reported as the reciprocal serum dilution required to achieve 50% maximal binding. Participants in the active group were stratified based on whether they had **(B)** HPV16 or **(C)** HPV31 NAb at baseline (D-1 NAb+) and their AAVLP-HPV antibody titers were compared. Sample sizes were n = 4 for Day −1 HPV16 NAb+ and n = 10 for Day −1 HPV16 NAb−, and n = 6 for Day −1 HPV31 NAb+ and n = 8 for Day −1 HPV31 NAb−. Mean ± SEM shown.
